# Towards a Hierarchical Strategy to Explore Multi-Scale IP/MS Data for Protein Complexes

**DOI:** 10.1371/journal.pone.0139704

**Published:** 2015-10-08

**Authors:** Joachim Kutzera, Age K. Smilde, Tom F. Wilderjans, Huub C. J. Hoefsloot

**Affiliations:** 1 Swammerdam Institute for Life Sciences, University of Amsterdam, Amsterdam, The Netherlands; 2 Netherlands Institute for Systems Biology, University of Amsterdam, Amsterdam, The Netherlands; 3 Faculty of Psychology and Educational Sciences, KU Leuven, Leuven, Belgium; 4 Faculty of Social and Behavioural Sciences, Leiden University, Leiden, The Netherlands; Medical University of South Carolina, UNITED STATES

## Abstract

Protein interaction in cells can be described at different levels. At a low interaction level, proteins function together in small, stable complexes and at a higher level, in sets of interacting complexes. All interaction levels are crucial for the living organism, and one of the challenges in proteomics is to measure the proteins at their different interaction levels. One common method for such measurements is immunoprecipitation followed by mass spectrometry (IP/MS), which has the potential to probe the different protein interaction forms. However, IP/MS data are complex because proteins, in their diverse interaction forms, manifest themselves in different ways in the data. Numerous bioinformatic tools for finding protein complexes in IP/MS data are currently available, but most tools do not provide information about the interaction level of the discovered complexes, and no tool is geared specifically to unraveling and visualizing these different levels. We present a new bioinformatic tool to explore IP/MS datasets for protein complexes at different interaction levels and show its performance on several real–life datasets. Our tool creates clusters that represent protein complexes, but unlike previous methods, it arranges them in a tree–shaped structure, reporting why specific proteins are predicted to build a complex and where it can be divided into smaller complexes. In every data analysis method, parameters have to be chosen. Our method can suggest values for its parameters and comes with adapted visualization tools that display the effect of the parameters on the result. The tools provide fast graphical feedback and allow the user to interact with the data by changing the parameters and examining the result. The tools also allow for exploring the different organizational levels of the protein complexes in a given dataset. Our method is available as GNU-R source code and includes examples at www.bdagroup.nl.

## Introduction

Proteins in a living cell interact and build functional units to play their role in the cellular machinery [1–4]. These units, called protein complexes, carry out many functions in the cell, and comprehending their composition is the key to understanding the cellular machinery in greater detail. Protein complex formation takes place at different levels of interaction [5, 6]. At a low interaction level, individual proteins bind together to build complex cores, stable modules that are the building blocks of protein complexes. A protein complex itself represents the next higher interaction level and is assembled from one or more cores. Different protein complexes that use the same core are possible. At still higher interaction levels, proteins build larger functional units that can consist of physically bound complexes or complexes that interact transiently [7].

Characterizing protein complexes in a cell sample is still a delicate task, although the research field has progressed considerably and proteins can now be identified and quantified using high–throughput methods, such as immunoprecipitation followed by mass spectrometry (IP/MS) [3, 8, 9]. In one IP experiment, a specifically designed antibody molecule (bait) is used to isolate its target protein (prey) from the sample, together with the proteins that are bound to the target. The proteins are quantified and identified with mass spectrometry (MS) [3, 10]. IP/MS experiments for different target proteins in the same sample result in different sets of detected proteins. Combined results from such different IP experiments contain two types of information, namely the occurrence and the abundance of each protein in each experiment.

**Fig 1:**
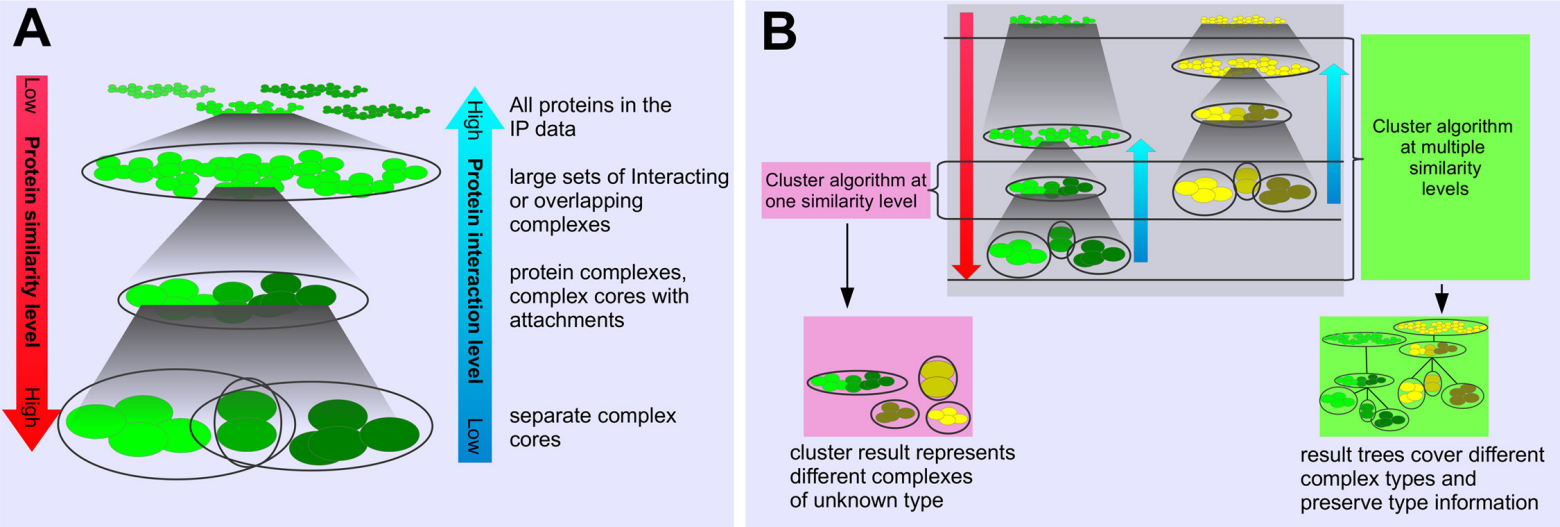
**A**: Proteins interact at different levels, from the low level of stable complex cores to the high level of temporarily interacting complexes. The different interaction types lead to different protein similarity levels in the context of the IP/MS data. Proteins of complex cores have a high similarity, while proteins of higher interaction levels have a lower similarity to each other. **B**: Two independent protein assemblies (depicted as green and yellow) and how they split in lower interaction levels. Protein complexes at different interaction levels can have the same similarity level. The clusters from a clustering method at one level (left) can represent complexes of different types for this reason, and it is unclear what each cluster represents. Our strategy (right) captures complexes at different similarity levels for this reason and creates trees that allow for predicting the interaction level.

In the context of IP/MS data, proteins are considered “similar to each other” when they occur together across experiments and have similar abundance values. This often holds for proteins that build a complex together; however, the different characteristics of protein complexes lead to different similarity levels of their proteins in the data. This phenomenon is shown in Fig 1A. Firmly bound proteins within a complex core are very similar to each other because they occur together in similar abundance throughout large parts of the IP/MS data. A complex that consists of this core and different attachments appears as one set of proteins that are less similar than the core proteins. Interacting complexes can give rise to a single large set of proteins with relatively low similarity.

The similarity of interacting proteins in IP/MS data makes it possible to detect protein interaction and complexes with clustering tools [3], which create clusters (sets of proteins) that represent the complexes. Gavin et al. and Krogan et al. presented large–scale IP/MS datasets from yeast and introduced methods to detect pairwise protein–protein interactions in their datasets [11–13]. Their datasets have been widely used for comparing further methods that find protein–protein interactions or protein complexes [14–20], but there is no consensus on which method works best, and most publications do not distinguish between the different complex types. Malovannaya et al. showed in their large scale human IP study that protein complex cores can be found using an intuitive method that is based on searching for protein sets with high co-occurrence and reciprocal similarity [21, 22]. The ideas from these publications were generalized in Kutzera et al. [23] and it was shown that the method works on datasets of different size and structure.

Different protein complexes at a specific interaction level, such as complex cores, do not always appear at the same similarity level in IP/MS data (Fig 1B). Most complex detection tools analyze the data at a specific (and often unknown) similarity level, and thus, their clusters may represent different types of complexes. To our knowledge, there is no clustering method that provides information about the interaction level or the similarity level of the found complexes. This complicates tuning the parameters of such methods to find a specific complex type and furthermore, hampers the interpretation of the results considerably.

We overcome these limitations with a new strategy that takes into account that protein complexes exist at different interaction and similarity levels. Our strategy is a hierarchical version of the 4N algorithm from [23] that we name HC4N (Hierarchical clustering using 4N). Unlike other complex finding tools, HC4N captures complexes at different similarity levels from low to high and creates hierarchical tree structures of clusters that reveal the interaction levels of the complexes. Moreover, unlike classical hierarchical clustering, HC4N allows for cluster overlap at each level of the hierarchy.

Like previous methods, HC4N assigns proteins that are similar to each other to clusters. In addition, our method provides information about why proteins were clustered together and where these clusters can be split into smaller clusters of more similar proteins that represent complexes at a lower interaction level. This divide-and-conquer strategy makes it possible to capture different interaction levels from large sets of complexes down to the stable cores each complex is built of. New graphical result representation methods are part of HC4N. They visualize at which similarity level complexes are found in the data and make predictions about their interaction level possible. They also help in adjusting the method’s parameters to fit different IP/MS datasets and finding different types of complexes.

## Materials and Methods

### Datasets

Several IP/MS datasets from yeast and humans are used to study the properties of our method and for comparison with other methods. Together with the IP/MS data, species–specific reference protein complexes are needed for the evaluation. Table 1 gives an overview of all IP/MS datasets and their types.

**Table 1:** IP/MS datasets used for the analyses.

Dataset	# IPs	# proteins	Type of data	Availability
Krogan2004	153	483	0/1	bioconductor
Krogan2006	2264	5323	0/1	bioconductor
Gavin2006	1752	2551	0/1	bioconductor
Gavin2006-SOI	63	39	0/1	G.2006 subset
Malovannaya	3290	11485	abundance	by authors
Malovannaya-SOI	1167	74	abundance	Malov. subset

We used the large–scale yeast IP/MS dataset that was presented by Gavin et al. [11] and two IP/MS datasets from Krogan et al. [12, 13]. We refer to these datasets as “Gavin2006,” “Krogan2004” and “Krogan2006.” A subset from Gavin2006 (called “Gavin2006-SOI”) is created using HC4N (see the detailed analysis in the results section). As a complex reference for the yeast datasets, we used the well-established cyc2008 [7] catalog. This database contains an up–to–date reference set of 400 annotated yeast protein complexes and was previously used in other publications to evaluate complex prediction methods [17–19].

The human IP/MS dataset of Malovannaya et al. [22] is the largest dataset in our analysis, and we refer to it as “Malovannaya.” From this dataset, we also derived a subset of certain proteins for which very precise information about complex–complex interactions is available. These proteins belong to the interaction complexes of “Mediator” (MED, [24]), “Integrator” (INT, [25]) and “RNA-Polymerase” (POL), which are described in two Malovannaya publications [21, 22]. We created an IP/MS subset (”Malovannaya-SOI”) that contains all these proteins and all corresponding IPs.

A satisfying reference for human protein complexes is still difficult to obtain. The best–known database for human interactions is CORUM [26]. However, complexes from CORUM are mainly functionally annotated, and unlike the complexes in cyc2008, they overlap highly due to redundancy in existing annotations [27]. Therefore, not every complex configuration from CORUM appears in the IP/MS data, which makes it difficult to use the database as a protein complex reference. For the Malovannaya dataset, we used several sets of complexes from the Malovannaya publications as reference for this reason. A detailed list can be found in S1 Table. Information about the complex–complex interactions were obtained from the same publications.

### The HC4N method

HC4N is based on the 4N method [23], which we will explain briefly here and in detail in S1 Text. 4N finds clusters called “near neighbor networks” in the IP/MS data. They are sets of similar proteins in terms of high pairwise co-occurrence, high set–wise completeness (all proteins in a near neighbor network co-occur highly with each other) and similar abundance. Each protein is assigned to many near neighbor networks by the 4N method.

Three global threshold parameters, one for each of the three above–mentioned similarity types, are used to set the strictness for calculating the near neighbor networks. The co-occurrence threshold parameter denotes in how many IPs two proteins need to co-occur relative to the number of IPs where any of them occur. The set–wise completeness parameter denotes how exclusive a near neighbor network needs to be. A low threshold allows for overlapping near neighbor networks, while a high threshold produces near neighbor networks that occur exclusively in this configuration. The abundance similarity is defined by the cosine similarity between two proteins.

The 4N method can set the thresholds for co-occurrence and set–wise completeness automatically to the strictest setting at which no proteins are lost, and it also returns the values as user feedback. The abundance similarity parameter is of minor importance (and not applicable for 0/1 data) and set by hand to 40 in all experiments. At low strictness settings, proteins with at least low similarity are assigned to large clusters that then represent a high protein interaction level. Proteins with high similarity are assigned to clusters when 4N is applied with high strictness settings, and they represent a low interaction level. Clusters that overlap by a certain percentage (usually 50%) are joined to larger clusters, creating the final result of 4N.

**Fig 2:**
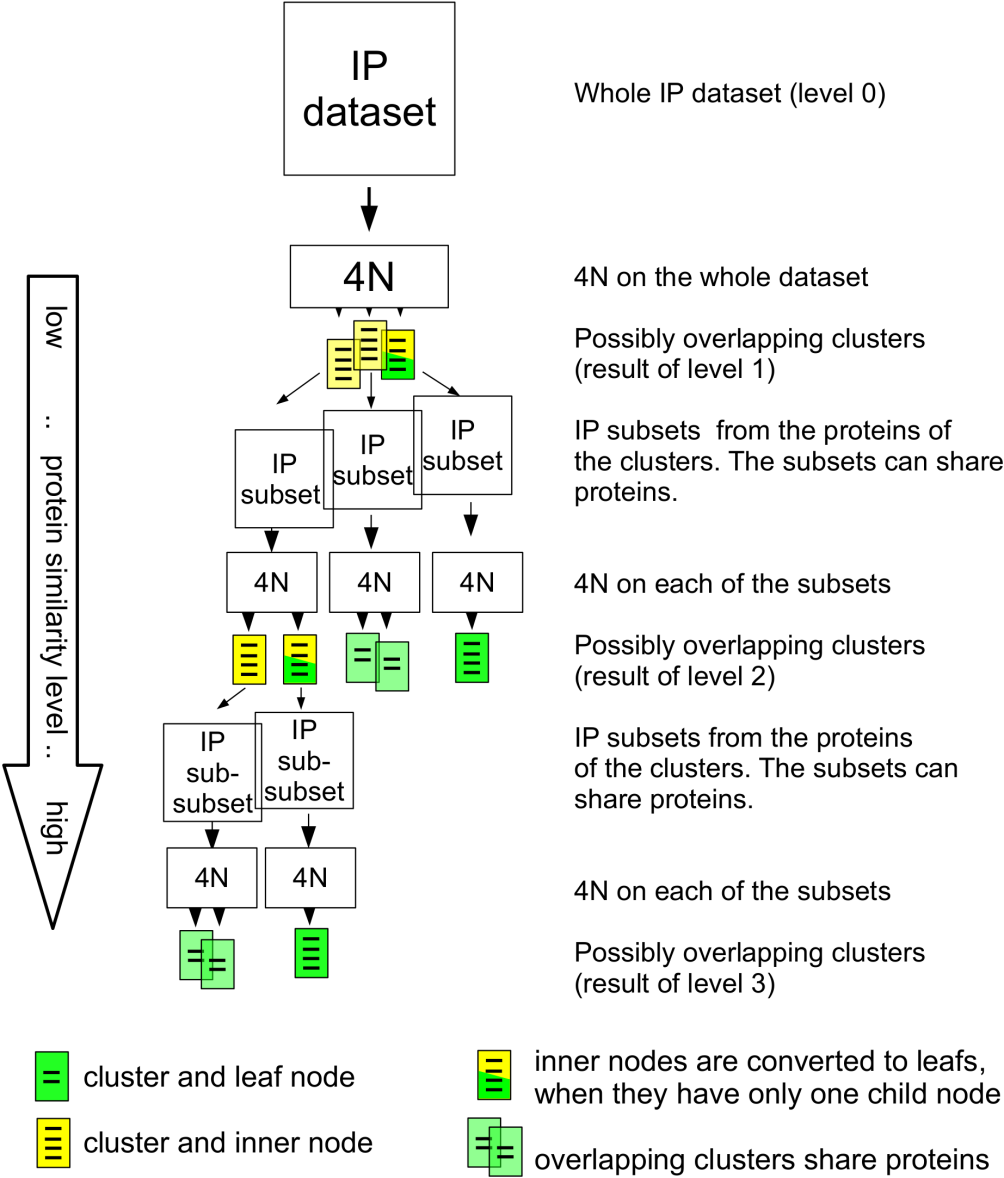
General overview of the HC4N strategy. The IP/MS dataset is analyzed with 4N to create the clusters of level 1. The dataset is split up into subsets where each subset contains all proteins from a level–1 cluster. 4N is applied to each of the subsets to create level 2. The procedure is repeated to create levels 3 and above, until no further splits are possible.

The HC4N strategy uses the ability of 4N to capture clusters at different similarity levels. It starts by applying 4N with low strictness to the IP/MS dataset. The resulting clusters are at level 1 in the result hierarchy tree; see Fig 2. They represent remotely interacting proteins at a high interaction level. For each of the (possibly overlapping) protein clusters, HC4N creates an IP/MS subset of its proteins by extracting the proteins and all IP experiments where they occur. Next, these subsets are analyzed using 4N with higher strictness. This second set of clusters represents level two in the result tree: a higher protein similarity level and a lower protein interaction level. The clusters are used again to create smaller IP/MS subsets, and again, they are analyzed with a stricter setup of 4N. This continues until the clusters cannot be split further.

The parameters of HC4N are set manually for the first level (see the result section for details). A manual setting of the parameters for all levels would be impossible as the total number of parameters can get very large. Therefore, the parameters for the higher levels are set automatically to the highest values where all proteins from the current subset are assigned to at least one cluster. As each subset is divided into smaller subsets of more similar proteins in a step, the HC4N strategy automatically captures a higher protein similarity level than before. Clusters of one step can have proteins in common. This facilitates, for example, that a core with different attachments can appear as a different cluster for each combination of core and attachments.

The result of HC4N is a tree–structured graph where the root node (level 0) contains all proteins in the IP/MS dataset. The root node has a child node for each level–1 cluster. Each node contains a cluster that is calculated from the subset of the previous node in the hierarchy. A node is a leaf when its cluster is not split further or an inner node with child nodes when its cluster is split into smaller clusters. The protein similarity of each cluster is judged using the minimum co-occurrence of its proteins. A cluster with a low co-occurrence represents proteins with low similarity at a high interaction level. Clusters with a high co-occurrence represent a low interaction level. Child nodes of an inner node have a higher co-occurrence than their parent node, as they were built by splitting the parent node into proteins of higher similarity.

**Fig 3:**
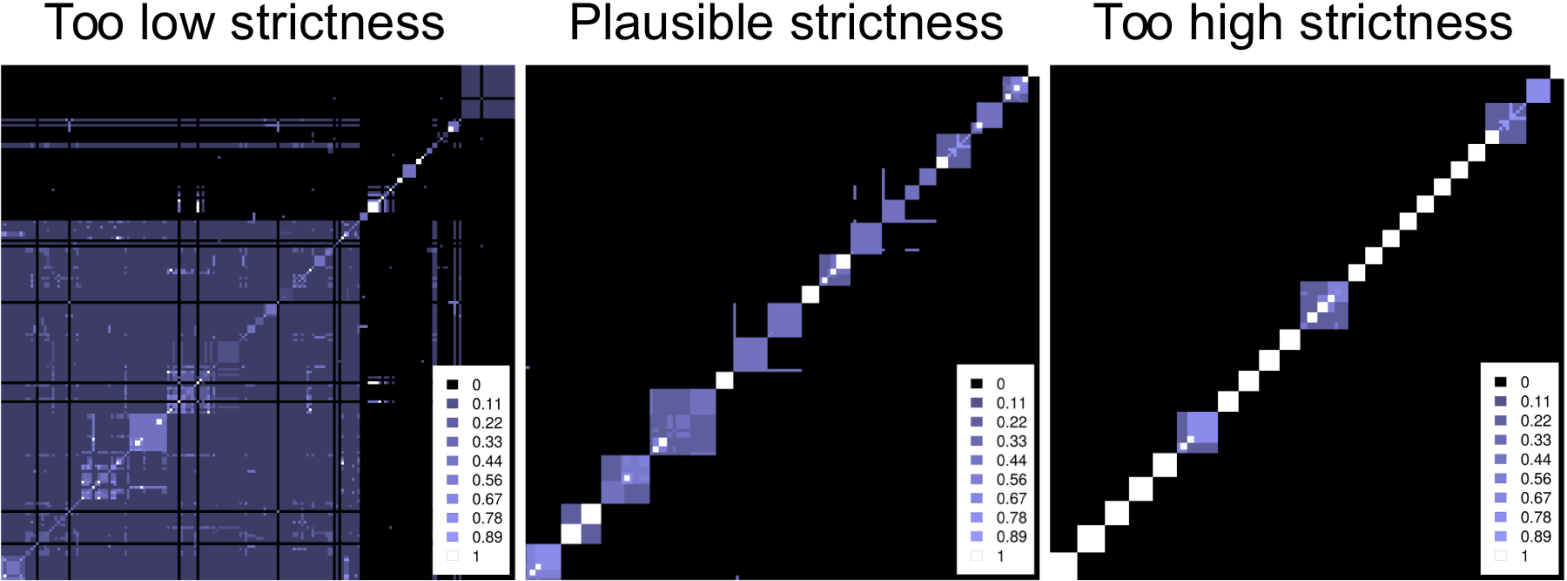
Example hierarchical cluster plots for different co-occurrence thresholds at HC4N level 1. The plots are small cutouts from the analysis of the Krogan2004 dataset. Left: The threshold is set too low with 0.125. Randomly co-occurring proteins lead to large, highly overlapping clusters, which do not represent protein complexes. At a higher threshold of 0.35, the clusters overlap less, and possible complexes and cores are visible. At a too–high threshold of 0.6, the clusters represent mostly complex cores, and their relation to each other is not visible. HC4N sets the set–wise completeness threshold automatically to 0.64 in all three examples.

Visualization of the HC4N result is crucial for interpreting the results and can be done in different ways. One way is the “hierarchical cluster plot” (HC-plot, see Fig 3), a heatmap–type diagram showing all proteins vs. each other. The HC-plot visualizes clusters at different co-occurrence levels. It shows which proteins are in a cluster together with a certain co-occurrence and whether this cluster is split into smaller clusters of higher co-occurrence. A cluster at a low protein similarity level occurs as a large square with a deep blue color in the HC plot. When the cluster has child clusters at a higher similarity level, they appear within that square as brighter colored, smaller squares. The plot also shows at which similarity level the clusters cannot be split further. Details about creating the HC-plot can be found in S1 Text. The HC-plot does not visualize every detail of the HC4N result; however, it gives insight into the different similarity levels, especially for large datasets, which are hard to visualize. It also helps in selecting the strictness for the first level of HC4N.

**Fig 4:**
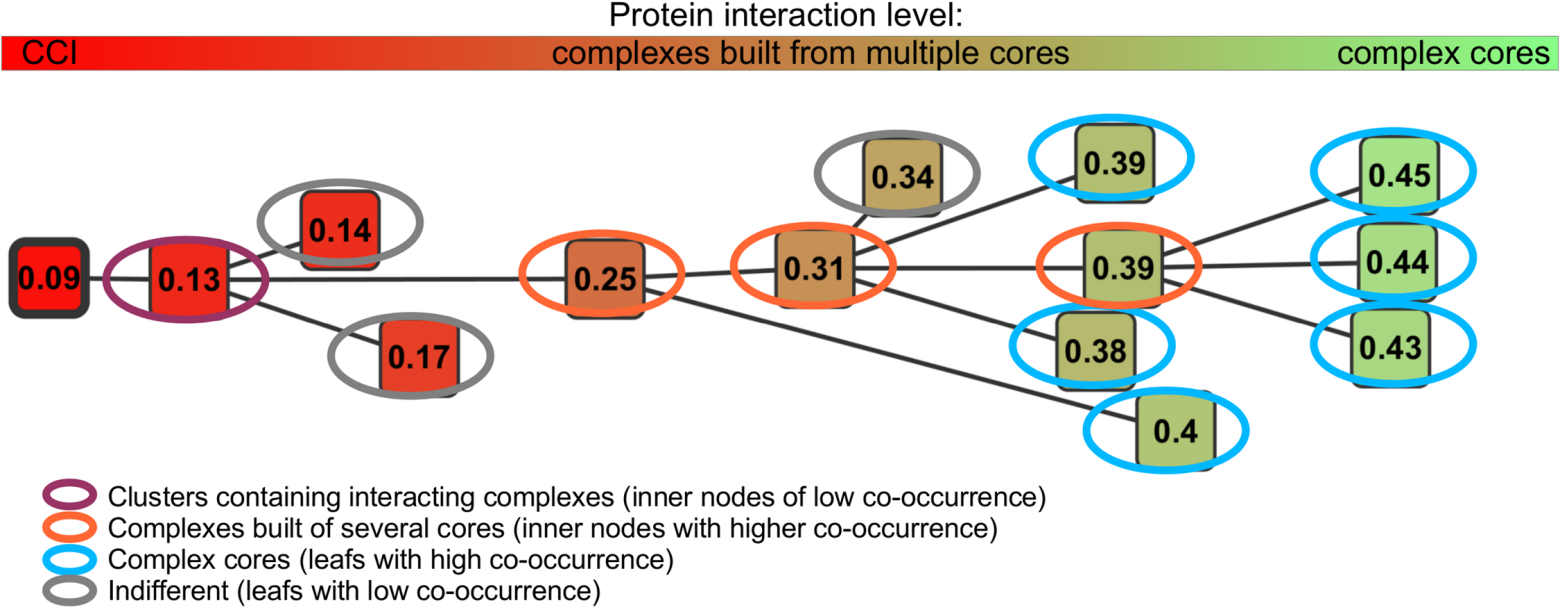
Example HC4N result tree. For clarity, the co-occurrence is displayed in each node. Clusters with many proteins of low co-occurrence and with large child nodes indicate interacting complexes at the highest interaction level. Complexes built of several cores have a higher co-occurrence and leafs as child nodes. Leaf nodes with a high co-occurrence symbolize complex cores. Leaf nodes with low co-occurrence mostly do not represent complex cores, and their interpretation is not always univocal.

For a more comprehensive analysis, the tree–shaped graph can be directly visualized with the graph tool cytoscape [28], as shown in Fig 4. Each node in the cytoscape representation contains the proteins of one cluster, and the node color represents its co-occurrence value. The graph shows directly at which similarity level certain clusters exist and where they are split into smaller clusters. This visualization is especially suitable for small datasets.

**Fig 5:**
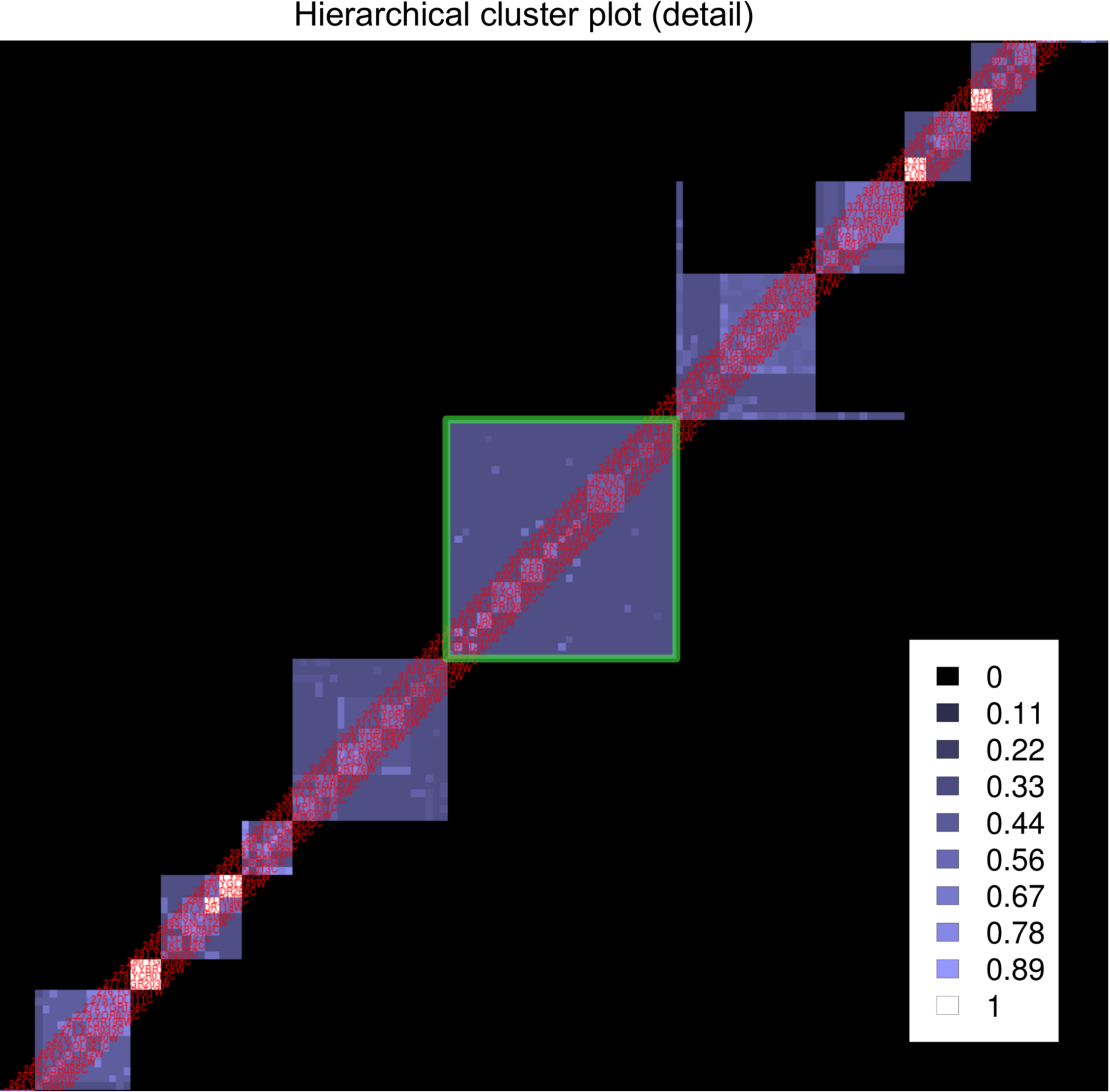
Extract from the HC–plot of Gavin2006 showing subsets of interest (SOIs). The green square frames the SOI of the three POL complexes. Black represents that two proteins are never in the same cluster, dark blue colors represent clusters with a low co-occurrence and bright colors represent clusters with a high co-occurrence.

Clusters with a low co-occurrence and with many child clusters of higher co-occurrence represent sets of overlapping or interacting protein complexes and should receive special attention. We call such clusters subsets of interest (SOIs). They appear in the HC-plot as large squares with complex inner structures as shown in Fig 5. HC4N, with parameters optimized for the large–scale dataset, might not reveal the correct inner composition of each SOI. For this reason, the dataset derived from a SOI should be treated as a new (small) dataset and analyzed again with HC4N.

### Other IP/MS analysis methods

SOIs are small, which makes it possible to analyze them with other methods for which the full dataset would be too large. We will discuss the SOI analysis with the methods Biclust [29], HICLAS [30] and apComplex [31] in this publication. Biclust [29] is used for inducing highly overlapping protein complexes from dense small–scale IP/MS datasets. The method is probabilistic, and as it needs many iterations to give reliable results, it is very computationally intensive. Biclust can process both occurrence and abundance data.

The HICLAS (HIerarchical CLASses analysis) [30, 32] algorithm has not yet been used for IP/MS analysis, but its underlying model fits the expected effects of overlapping clusters on pure 0/1 data and it was tested for that reason. An HICLAS model with *K* clusters creates *K* protein clusters and *K* IP clusters. In the model, an *I* × *J* protein by IP dataset *D* is approximated by a model matrix *M* of the same size. *M* is composed as *M* = *A* ⊕ *B*, where *A* is a *I* × *K* binary matrix with *A*_*i*, *k*_ denoting whether protein *i* is in protein cluster *k*, *B* is a *J* × *K* binary matrix with *B*_*j*, *k*_ denoting whether IP *j* is in IP cluster *k* and ⊕ is a binary matrix multiplication operator where each result > 0 is set to 1, for example, 1+1 = 1. HICLAS minimizes the residuals function *f* over the matrices *A* and *B*, where *f* denotes the sum of squared differences between the model matrix *M* and data matrix *D* as

$$\begin{array}{c} f\left(A,B\right)=\sum_{i=1}^{I}\sum_{j=1}^{J}{\left(D_{i,j}-M_{i,j}\right)}^{2}. \end{array}$$ (1)

We applied HICLAS in our tests with different numbers of clusters and examined the residuals of each analysis to find the optimal number of clusters. We used the possibility to weight negative residuals differently than positive residuals [33].

The method apComplex [14, 31] uses a local modeling algorithm on the bait-prey interaction graph to reconstruct possible complexes in pure occurrence data and was previously used for analyzing the yeast datasets.

### Cluster quality assessment

The tree–shaped graphs from our HC4N method contain more information than just the clusters themselves; however, no comparison method that takes this additional information into account is available. To make the comparison possible, we removed the tree information from the result and joined clusters that were overlapping by more than 60%. The same joining step was applied to the results of the other methods for a fairer comparison. This joining step increased the quality of all methods because they often produce numerous, very similar small clusters that, when joined, represent the reference complexes better.

We used the method by Brohée and van Helden [34] to evaluate our results. The method is capable of measuring how accurately a set of reference complexes is predicted by a set of clusters, and it has already been used to assess complex predictions in other studies [17, 18]. Three quality measures are provided by the method: sensitivity, positive predictive value (PPV) and accuracy. The sensitivity is the fraction of proteins from the reference complexes that are found in the predicted clusters; the PPV is the fraction of proteins from the predicted clusters that belong to the reference complexes. From sensitivity and PPV, the accuracy is calculated as the square root of their product. For a set of predicted clusters and a reference complex set, an accuracy of 1 is reached when each reference complex perfectly matches one of the clusters.

One shortcoming of this method is that the accuracy does not decrease when a prediction method produces too many clusters that contain proteins from the reference complexes, as just the best matching cluster for each reference complex is taken into account. Hence, a score called separation is provided by the method in addition, denoting how many predicted complexes represent one reference complex. The separation score is 1 when each predicted complex covers exactly one reference complex.

In our comparison, not all analysis methods could be applied to all datasets. For apComplex, the large datasets do not produce results due to memory problems when running on a PC with 12 gigabytes of memory. Both Biclust and HICLAS did not produce results on the large sets within a reasonable amount of time.

## Results

The HC4N performance depends on optimal parameters for level 1. To find these parameters, we initially allowed HC4N to create the level–1 clusters with automatic setup where the thresholds for co-occurrence and set–wise completeness are set as high as possible so that each protein is still assigned to at least one cluster.

We examined the HC-plot for the result to determine at which protein co-occurrence level the first clusters appear. When the clusters were too large, we set the co-occurrence threshold slightly higher than before, and when they were overlapping too much, we set the set-completeness parameter higher. For the SOI analyses, we have set the co-occurrence threshold and set-completeness parameter slightly lower than in the automatic setup to detect the interaction level of complex–complex interactions (see below). A scheme for how to use HC4N is located in S2 Text and details for each large–scale analysis, including Figures, are in S3 Text.

The results are summarized in Table 2. HC4N gains good sensitivity and PPV for most datasets. The separation values are between 0.2 and 0.42, which is acceptable but shows that HC4N, like most methods, tends to create slightly too many clusters. A better separation value would be achieved by joining the clusters with a lower threshold at the cost of a lower specificity.

**Table 2:** Summarized results of HC4N on all tested datasets.

Dataset	Sensitivity	PPV	Accuracy	Separation	Runtime
Krogan2004	0.8	0.70	0.75	0.42	2 minutes
Krogan2006	0.5	0.72	0.6	0.22	8 hours
Gavin2006	0.78	0.68	0.73	0.36	30 minutes
Malovannaya	0.84	0.90	0.87	0.21	1 day
Gavin2006-SOI	0.88	0.58	0.72	0.27	2 minutes
Malovannaya SOI	0.99	0.78	0.88	0.21	2 minutes

Table 3 compares our accuracy with other methods. We obtained the scores for Biclust, HICLAS and apComplex by applying the methods ourselves; the results for Wu et al. [17] and Cai et al. [18] were taken from the original publications. The table shows that our method has better accuracy in most cases. The dataset Krogan2004 is of low complexity, and previous methods already gained an accuracy of 0.72, which was still increased to 0.75 by HC4N. A more substantial improvement was reached for Gavin2006 (0.73 compared to 0.57). Krogan2006 is difficult to analyze, which is why previous methods scored below 0.5 and also why HC4N only achieves 0.6.

**Table 3:** Accuracy of HC4N in comparison to competing methods.

Dataset	Cai et al.	CACHET	apComplex	Biclust	HICLAS	HC4N
Krogan2004	NA	NA	0.72	0.6	NP	0.75
Krogan2006	0.4	NA	NP	NP	NP	0.6
Gavin2006	0.57	0.52	NP	NP	NP	0.73
Gavin2006-SOI	NA	NA	0.72	0.72	0.72	0.72
Malov. SOI	NA	NA	0.69	NA	0.75	0.88

The values for CACHET [17] and Cai et al. [18] were taken from the respective publications. We applied the other methods on all datasets. “NA” means that no value for this dataset was available in the corresponding original publication. “NP” means that we tried the method on the dataset but it was not possible to produce a result.

When compared to other methods, HC4N does not always yield a larger separation score (see S2 Table). In these cases, however, HC4N and the other methods give separation scores that are in the same range. The two SOIs deserve a more detailed analysis as they contain overlapping and interacting complexes. They are discussed below.

### Analysis of the Gavin2006-SOI dataset

The HC-plot from the Gavin2006 analysis shows subsets of interest, and we analyzed the green–framed SOI (see Fig 5) as an example. We assume for this demonstration that we do not know what type of complexes the cluster of the SOI contains. The subset Gavin2006-SOI contains all proteins from the cluster and all IPs in which any of the proteins occur.

**Fig 6:**
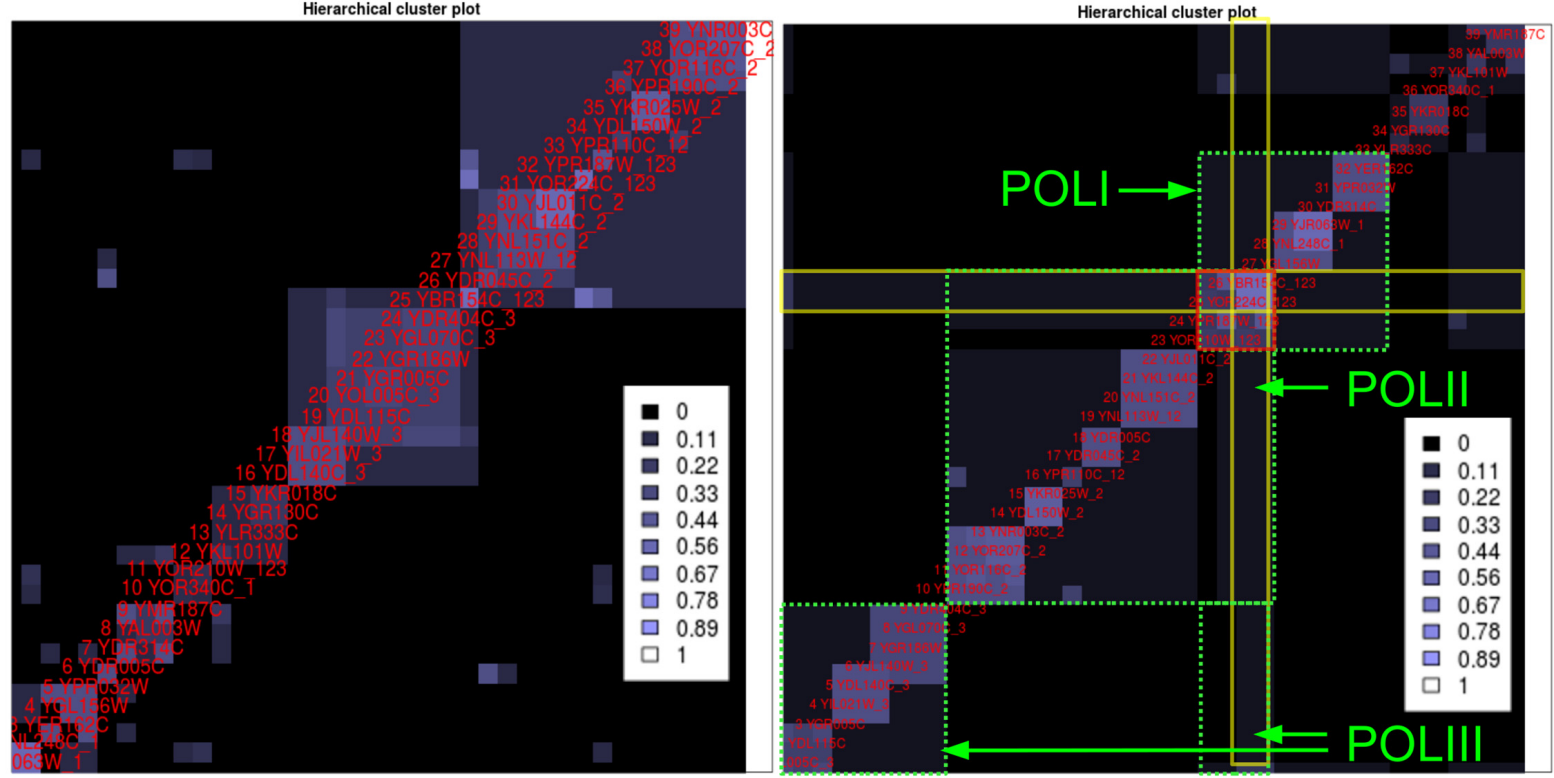
Hierarchical cluster plots for two different HC4N parameter settings on the Gavin2006-SOI dataset. The numbers at the labels (_1,_2,_3,_12,_123) show the POL-cluster assignments according to cyc2008. **Left**: Too–strict parameter settings (co-occurrence threshold 0.25, completeness threshold 0.4): The clusters are scattered and show low overlap. It is not clear which proteins are shared by how many complexes. **Right**: Lower parameters (0.2 and 0.5). The green frames show the three POL complexes as the HC4N result. The red square shows a characteristic pattern of four highly co-occurring proteins that in addition, partly co-occur with most other proteins (yellow frames). They are the shared proteins of the three complexes.

Analyzing the dataset starts by running HC4N with automatic setup and inspecting the HC-plot. The plot shows complexes but only a few connections between them, as shown in Fig 6, left side. We already know that all proteins have a certain degree of similarity because they were assigned to one cluster in the large–scale analysis. We conclude that the automatic parameters are too strict and not optimal for finding clusters with shared proteins. Therefore, the parameters are lowered until the new HC-plot (Fig 6, right) shows a characteristic pattern that denotes shared proteins. This pattern features proteins (which are the shared proteins) with a very high co-occurrence to each other and a high co-occurrence to many other proteins from different clusters. In the figure, the pattern contains YOR224C and YBR154C, which have a high co-occurrence to almost all proteins in the plot. Two other proteins (YOR210W, YPR187W) have a high co-occurrence to these two proteins. Three large clusters are shown in the HC-plot, and the two proteins appear in all of them. We can assume that the SOI contains (at least) three clusters that share the proteins from the pattern. The two other proteins that are similar to the shared proteins are likely to be shared as well.

A comparison with the cyc2008 reference shows that the three clusters represent the three POL complexes and that the four mentioned proteins are shared by them. The cluster result does not show that two additional proteins are shared between POLI and POLII, as they are exclusively assigned to POLII. Twelve SOI proteins are not in the cyc2008 reference. They were searched on string-DB (www.string-db.org) [35], a graph–based on–line protein interaction database. We included the interaction types “Co-occurrence” and “Experiments” into the string-DB result, but no genetic information. We found that ten proteins interact with the POL complexes to which they were assigned by HC4N. A network from string-DB showing all POL proteins and the additional proteins can be found in S1 Fig.

**Fig 7:**
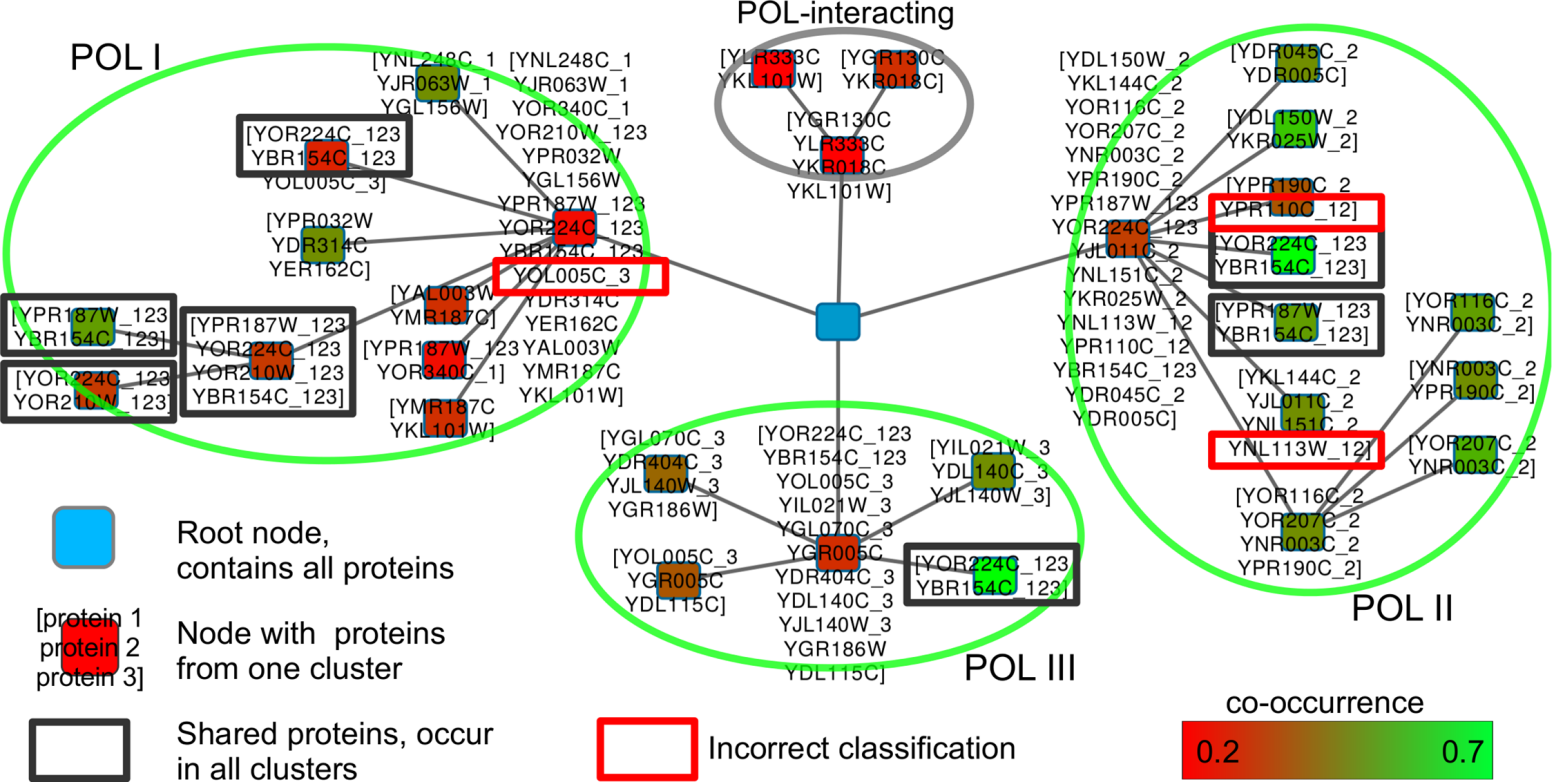
Network representation of the Gavin2006-SOI HC4N result. The green ovals indicate the three POL complexes as found by HC4N. The forth oval (gray) contains proteins that are known to interact with POL but were not in the cyc2008 reference. The protein name suffixes (_1,_2,_3,_12,_123) indicate the assignment to POLI-POLIII from the reference. The four shared proteins from the characteristic pattern (framed in dark gray) occur in all complexes and together multiple times.

The cytoscape visualization of the result graph (Fig 7) confirms the findings. It shows four clusters, of which the three largest clusters represent the three POL-complexes. The two proteins (YOR224C and YBR154C) appear in all clusters, and therefore we can assume that YOR224C and YBR154C interact with all complexes. The two proteins also appear together multiple times with YOR210W and YPR187W, which denotes that all four proteins play an important role in all three POL complexes.

The SOI was also analyzed with apComplex, BICLUST and HICLAS. ApComplex creates more than 200 clusters, leading to an accuracy of 0.76 but very poor separation of 0.05. After joining the clusters, a still–good accuracy of 0.72 at a now–good separation of 0.34 is reached. In all cases, apComplex misclassifies several proteins.

Too many and too–small clusters are built by BICLUST, which represent the POL complexes only partly and did not show the special role of the shared proteins. HICLAS was better able to capture POL but requires the cluster number as prior knowledge. None of the methods provides information about the interaction level of the clusters. A graphical comparison of the results between HC4N, BICLUST and HICLAS can be found in S4 Text.

### Analysis of the Malovannaya-SOI dataset

**Fig 8:**
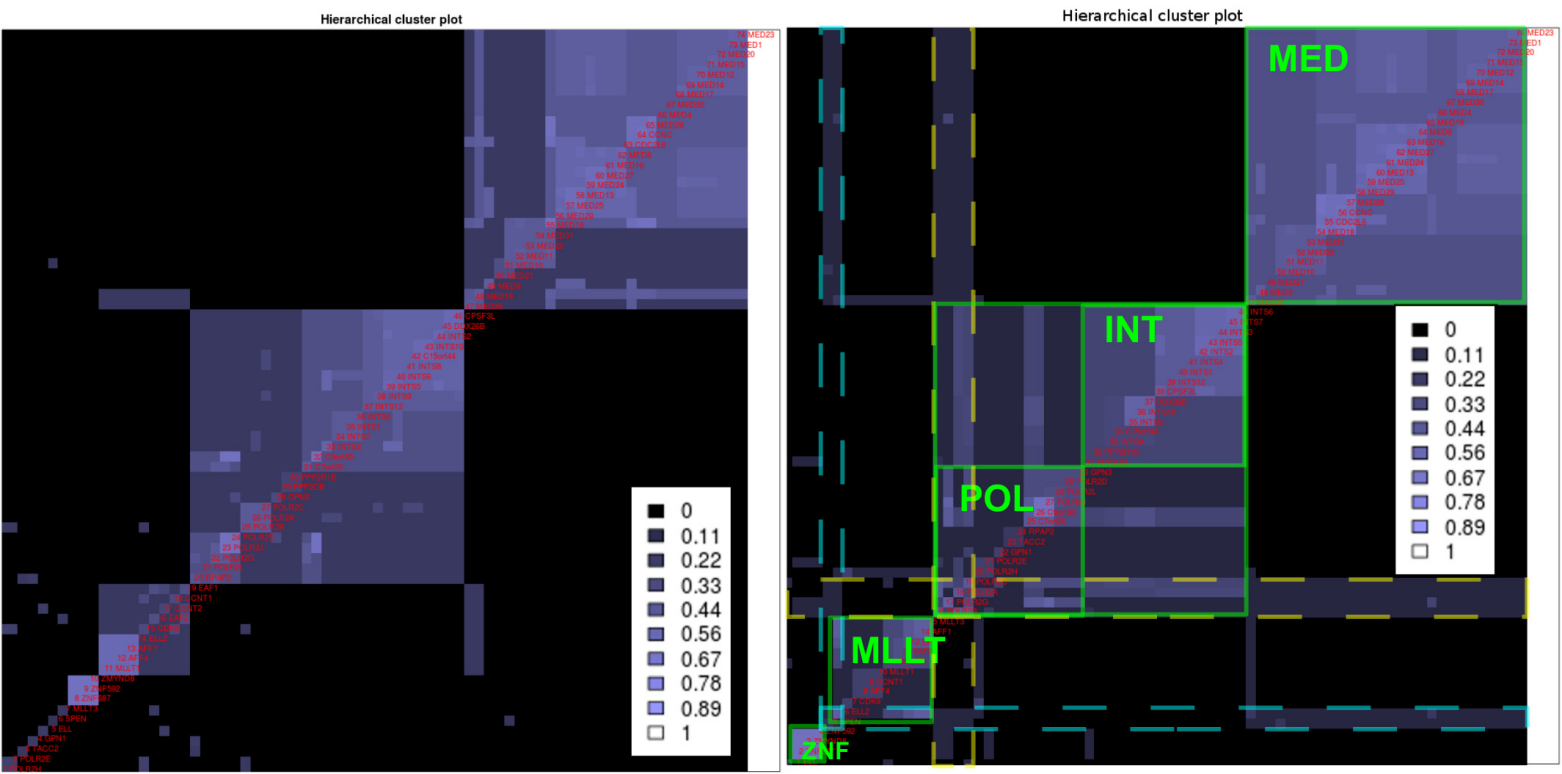
Hierarchical cluster plots for the Malovannaya SOI. **Left**: With too–strict automatic thresholds (co-occurrence 0.09, completeness 0.6), correct clusters are visible but not the connections between them. **Right**: Lower parameters (0.05 and 0.5) create patterns indicating shared proteins. Two large and two small clusters are visible, framed by the thick green squares. MED appears as a separate cluster, while INT and POL appear together as dense subsets of one cluster. POL and INT can build a more stable complex together than POL and MED. One pattern (framed in yellow) contains POLR2 A/B/C/G, which are important interactors within both the POL-INT and the POL-MED complex. ELL2 and SPEN (framed in cyan) comprise another pattern and are important for the interaction between MLLT and the MED complex.

HC4N is applied with automatic settings, and the HC-plot is examined (Fig 8, left). Several clusters are visible but only a few connections between them. We know based on the protein selection that their clusters interact, and now we want to explore these interactions in detail. We set the strictness for level 1 low enough such that the HC-plot shows the characteristic patterns indicating complex interactions. The new plot (Fig 8, right) shows two large and two small clusters of different structure. While one large cluster is dense and with high co-occurrence, the other has a lower co-occurrence and two dense subclusters of more similar proteins are visible within it. Two of the characteristic patterns, one with two and one with four proteins, occur across several but not all clusters.

We conclude from the plot that the large cluster of lower co-occurrence is built from two closely interacting subcomplexes, one containing the proteins of the four-protein pattern that facilitates the interaction between the two subcomplexes. One subcomplex also interacts separately with the large dense complex but less closely. The two-protein pattern indicates interaction of the large dense complex with the two other small complexes.

The comparison with the reference shows that the MED complex is represented by the large dense cluster and that the complexes POL and INT are the two subcomplexes of the other cluster. The proteins POLR2A/B/C/G build the four-protein pattern, and it is known that they facilitate the interaction between POL and INT as well as between POL and MED. It is also known that POL and INT build a complex together, while POL and MED interact more transiently. The two-protein pattern includes ELL and SPEN, which connect the small complex around MLLT and ZNF to MED but not to INT.

All conclusions agree with the information from [22] and [36]. We also created the network representation, see S5 Text. It allows the same conclusions and shows in a clearer manner which protein interaction level is represented by which similarity level. MED and INT are individually assigned to POL (but not to each other) in higher interaction levels, and the complexes separate in the lower levels. The same holds for the small complexes that interact with MED. In this specific dataset, the protein co-occurrence is relatively low, even within POL, MED and INT, but still higher relative to the complex–complex interaction forms POLR-MED and POL-INT.

The dataset was also analyzed with apComplex and HICLAS (see S4 Text). We were not able to run Biclust because the dataset is too large. As with the other SOI analysis, the methods were not able to capture the complexes correctly and created too many too–small clusters. None of the methods is designed to uncover the different levels of interaction between the complexes, and from their results, it is not clear which interaction level their clusters represent.

### Discussion and Conclusions

Finding protein complexes in IP/MS data is a difficult task. Protein complexes can be found at different organizational levels in IP/MS data, and these levels must be explored together. The task is twofold: i) finding the complexes at different levels and ii) visualizing the result in a way that makes the different levels visible. In essence, this is a data exploration and visualization problem, and we designed our method, HC4N, to address that problem.

Exploratory data analysis is a partly subjective task, e.g., by selecting parameters during analysis. While most software tools come with default parameters, understanding their effect on the result remains a problem. Our method not only supports automatic and manual parameter settings but also allows the user to retrace the effect of parameter changes with visual feedback. The change of parameters is very insightful because it enables exploring the different levels of organization of the protein complexes in a given dataset.

A major problem for all complex finding tools is noise in the IP/MS data, leading to many false positives. While HC4N cannot actively remove noise, its built–in visualization tools help in detecting noise. In noisy data, HC4N will find many small, possibly false-positive clusters at low co-occurrence levels when applied with automatic parameters. Detected true-positive clusters may not have a fundamentally higher co-occurrence than the false positives. This leads to characteristic HC-plots without the typical clusters of high co-occurrence that appear when analyzing low-noise datasets. The behavior is demonstrated with examples in S3 Text.

We have shown in this manuscript that protein complexes occur at different interaction and similarity levels, even in the same IP/MS dataset. Our new method, HC4N, is able to find complexes of different types and has been validated thoroughly using several datasets and comparisons with existing methods. The philosophy behind HC4N is to provide an interactive exploratory tool for analyzing IP/MS data that can be used (and tuned) by the biologists.

## Supporting Information

S1 Table Reference complexes for the Malovannaya datasets. Reference from Malovannaya et al..
(PDF)

S2 Table HC4N separation scores. Separation scores of HC4N in comparison with the other methods.
(PDF)

S1 Text 4N, HC4N, HC-plot, pseudocode. Details for 4N, HC4N and the hierarchical cluster plot. pseudocode for all modules.
(PDF)

S2 Text General HC4N strategy. Usage and parameter selection strategy for HC4N.
(PDF)

S3 Text HC4N analyses. HC4N analyses on the large–scale datasets.
(PDF)

S4 Text Biclust, apComplex, HICLAS. Analysis of Gavin2006-SOI and Malovannaya-SOI with Biclust, apComplex, HICLAS.
(PDF)

S5 Text Malovannaya-SOI HC4N graph. HC4N result graph for the Malovannaya-SOI analysis.
(PDF)

S1 Fig POL StringDB. All proteins of the Gavin2006-SOI dataset as string-DB network.
(TIFF)
