# Supplementary material for: Towards a Hierarchical Strategy to Explore Multi-Scale IP/MS Data for Protein Complexes
Source: PLoS One. 2015 Oct 8;10(10):e0139704. doi: 10.1371/journal.pone.0139704 (PMC4598013; doi:10.1371/journal.pone.0139704)
Supplement: S1 Table — Reference from Malovannaya et al.. (PDF) [file pone.0139704.s001.pdf]

Reference complex set from Malovannaya et al.

The complexes INT, POL, PPP, C**orf** and ZMYND were taken from Malovannaya et al. (2010), Figure 2. MED was taken from Fig. 3 of the same paper. The other complexes were taken from Figure 3 B in Malovannaya et al (2011).

INTS1 INTS2 INTS3 INTS4 INTS5 INTS6 DDX26B INTS7 INTS8 INTS9 INTS10 CPSF3L INTS12

POLR2A POLR2B POLR2C POLR2D POLR2E POLR2G POLR2H POLR2J POLR2L

PPP2R1A PPP2R1B PPP1CB PPP2CA PPP2CB

C12orf11 C15orf44 C7orf26 C9orf80

ZMYND8 ZNF592 ZNF687

MED1 MED4 MED8 MED12 MED13 MED14 MED16 MED17 MED18 MED20 MED23 MED24 MED25 MED26 MED27 MED29 MED30 MED19 MED15 MED28 MED9 MED22 MED11 MED10 MED21 MED31

CDC2L6 CCNC

TACC2 GPN1 GPN3 RPAP2

AFF1 AFF4 MLLT1 MLLT3 ELL ELL2 EAF1 EAF2

CDK9 CCNT1 CCNT2
