# Supplementary material for: Towards a Hierarchical Strategy to Explore Multi-Scale IP/MS Data for Protein Complexes
Source: PLoS One. 2015 Oct 8;10(10):e0139704. doi: 10.1371/journal.pone.0139704 (PMC4598013; doi:10.1371/journal.pone.0139704)
Supplement: S2 Table — Separation scores of HC4N in comparison with the other methods. (PDF) [file pone.0139704.s002.pdf]

Separation scores of HC4N in comparison with the other methods

The values for CACHET and Cai et al. were taken from the respective publications. The other methods were applied by us on all datasets. "NA" means that no value for this dataset was available in the corresponding original publication. "NP" means that we tried the method on the dataset but it was not possible to produce a result. For apComplex the large data sets are not producing results due to memory problems running on a PC with 12 gigabyte RAM. Both Biclust and HICLAS did not produce results within a reasonable amount of time.

Dataset	Cai et al.	CACHET	apComplex	Biclust	HICLAS	HC4N
Krogan2004	NA	NA	0.24	0.45	NP	0.42
Krogan2006	NA	NA	NP	NP	NP	0.22
Gavin2006	NA	NA	NP	NP	NP	0.36
Gavin2006-SOI	NA	NA	0.76	0.72	0.72	0.27
Malovannaya-SOI	NA	NA	0.14	NP	0.60	0.26
