# Supplementary material for: Towards a Hierarchical Strategy to Explore Multi-Scale IP/MS Data for Protein Complexes
Source: PLoS One. 2015 Oct 8;10(10):e0139704. doi: 10.1371/journal.pone.0139704 (PMC4598013; doi:10.1371/journal.pone.0139704)
Supplement: S1 Text — Details for 4N, HC4N and the hierarchical cluster plot. pseudocode for all modules. (PDF) [file pone.0139704.s003.pdf]

4N, HC4N, Hierarchical cluster plot

4N (Kutzera et. al, 2013) is based on the method 3N (Malovannaya et. al, 2010) and we used the terminology (NNN, core complex) of this publication. 4N consists of three steps, finding near neighbor networks (NNNs) for each protein, creating core complexes from the NNNs, and joining the core complexes to create the cluster result. Pseudocode for all steps are in the next section. The NNN of a protein contains all proteins that have a high co-occurrence (defined by the binary jaccard coefficient) and a low cosine distance (High similarity of abundance values across the samples) to this protein. The core complex for a specific protein is a subset of the proteins from its NNN. This subset contains all proteins, that have a similar reciprocal near neighbor network.

Core complexes that overlap by more than a certain threshold are joined. We define the overlap between two core complexes as the relative number of proteins in the smaller core complex that occur in the larger one. When the threshold is smallest, all core complexes that share at least one protein are joined, which leads to clusters that do not show any overlap. They represent sets of proteins that co-occur with proteins within the set but not with proteins in other sets. Higher thresholds create smaller sets of partly overlapping core complexes and at a threshold value of 1, complexes are only joined when the larger one contains all proteins of the smaller one. The joined core complexes are the cluster result of 4N.

Four parameters control 4N. U is the co-occurrence threshold for building the NNNs. Its biological meaning is that when two proteins are interacting, they occur together in IP data across the different samples rather than alone. The second parameter, C, is the threshold for the cosine distance and thus, only relevant when the data contains abundance values instead of occurrence values. Interacting proteins are expected to have similar relative values across samples and the cosine distance threshold tests for that. The third parameter (S) checks whether the proteins in a NNN of a specific protein have similar own (reciprocal) NNNs. Only those proteins are in the core complex of this specific protein. Parameter four (P) is the threshold for the overlap at which two core complexes are joined to one larger one. The cosine distance parameter has a range between 0 and 90, all other parameters between 0 and 1. In the default setup of 4N, U is set to the highest value at which every protein is assigned to at least one NNN. The default maximum cosine distance threshold is 40. S is set highest so that each protein is assigned to at least one core complex. Default threshold for joining the core complexes (P) is 0.5.

HC4N: The clusters from the first 4N run represent level 1 in hierarchical result. Each instance of HC4N receives clusters from the previous instance. It extracts subsets from the dataset where each subset contains all proteins from one of the clusters of the last instance. 4N is then applied with its automatic settings to each of these subsets. The result clusters of these 4N-runs are input for the 4N runs of the next level. When 4N found only one cluster in a subset, it is stored as leaf node in the result tree. When 4N found more clusters, it stores them as inner node in the result tree and starts a new instance of itself, using the 4N-result and the current IP subset as input.

The process continues until each subset cannot be split into smaller subsets anymore. The result is a set of tree structured graphs where each node in a tree contains a cluster that was calculated from the subset of the parent node. Nodes are leafs when the subset from the cluster cannot be split further or they can contain children nodes, which contain the clusters from the proteins of the current cluster. Each node also the smallest co-occurrence that any protein in the cluster has as maximum co-occurrence to another protein in the cluster. The value is larger or equal to the U with which the cluster was created because in an instance of 4N, a data subset falls into clusters at a certain U but the clusters itself can have a higher within-co-occurrence than this U.

High co-occurrence represent sets of proteins that build a stable complex, especially when the node is a leaf. Low co-occurrence in inner nodes show that the protein set splits up in smaller sets of higher connected proteins. Protein complexes that share proteins build an inner node with a relatively low co-occurrence together. Protein subsets of these complexes which are not shared by the other complexes appear as child clusters with a slightly higher value and the proteins that are shared by the complexes appear in an own child cluster with a very high value, as they appear together across most columns of the data subset.

The **hierarchical cluster plot** (HC-plot) is based on a matrix for all proteins vs. each other where each matrix cell is set to 0 first. The matrix cells for each pair of proteins that belong to the same level-one cluster is set to the co-occurrence value of this cluster. When a cluster has children clusters in the result tree of HC4N, the matrix cells of those children proteins are overwritten with the higher value of the child cluster. This continues until all child clusters of all level-one clusters are processed. The matrix is resorted using hierarchical clustering on the euclidean distance matrix (heatmap clustering). The HC-plot visualizes all levels of co-occurrence between the proteins. It shows, which proteins build clusters at certain co-occurrence values and whether those clusters are separated into groups of proteins with higher co-occurrence.

Pseudocode for all modules of 4N and HC4N

IP-matrix:

M x N matrix where each row stands for a protein and each column for an IP-Bait. Each matrix cell A $_{m,n}$ contains the abundance value of a protein *m* (or a 1 in occurrence data) in the IP-experiment with bait *n* or a 0 if the protein was not found in the experiment with this bait.

Function for calculating the near neighbor network (NNN)

Parameters:

U: co-occurrence threshold (automatically selected or external)
C: Cosine-distance threshold (external)

Input: IP-matrix

1 Create a M x M matrix "*coOcc*" with a 0 in each matrix cell
2 Calculate the Jaccard coefficient (see page bottom) for each combination of two proteins i,j and store it in *coOcc*$_{i,j}$
3 IF **U** is not given THEN
  3.1 Select the maximum value of each row in coOcc and set **U** to the smallest of these values that is > 0
4 Create a M x M matrix "*NNNs*" with a 0 in each cell
5 For each pair of protein indicies i,j:
  5.1 Set *NNNs*$_{i,j}$ to 1 where *coOcc*$_{i,j}$ is ≥ **U**
6 For each pair of protein indicies where NNN$_{i,j}$ is 1:
  6.1 calculate the cosine distance for the protein pairs i,j and *NNNs*$_{i,j}$ to 0 when the cosine distance is above the threshold **C**

Output: *NNNs*: M x M matrix for all proteins vs. each other. *NNNs*$_{i,j}$ is 1 if protein j is in the near neighbor network of protein i and 0 otherwise.

Function for calculating core complexes from the NNNs (CC)

Parameters:

S: NNN reciprocity threshold for building core complexes

Input: *NNN-matrix* where each row i stands for the near neighbor network of protein i

1 Create a M x M matrix "*CC*" with a 0 in each matrix cell
2 For each row i in *NNN*:
  2.1 Select the column indicies of all proteins that are in the near neighbor network of protein i and store them in a "*CC-candidate*" list
  2.2 Calculate the Jaccard coefficient (see end of this page) between row i and each row j from the NNN matrix that is in the *CC-candidate* list
  2.3 Keep the proteins in the *CC-candidate* list that have a Jaccard coefficient ≥ **S** and remove all other proteins
  2.4 Set CC in the row i for all column-indicies in the *CC-candidate* list to 1

Output: *CC*: M x M matrix for all proteins vs. each other. *CC*$_{i,j}$ is 1 if protein j is in the core complex of protein i and 0 otherwise.

Jaccard coefficient between two proteins i,j:
Let **K1** be the set of column indicies in the IP-matrix where the protein i occurs, **K2** the column indicies where j occurs.
Jaccard coefficient(i,j) = |**K1** ∩ **K2**| / |**K1** U **K2**|.

Cosine-distance between two proteins i, j in the *IP-matrix*:
Let **V1** contain the abundance values of protein i across all columns from *IP-matrix and* **V2** the abundance values of protein j across all columns. cosine-distance(i,j) = arccos(**V1 • V2** / ||**V1**|| **x** ||**V2**||).
IF |**K1** ∩ **K2**| == 1 THEN
  IF Jaccard coefficient(i,j) > 0.5 THEN cosine-distance(i,j) = 0
  ELSE cosine-distance(i,j) = 90

The if-clause is necessary because when two proteins share only one sample, it is unlikely that they are in the same complex when their Jaccard coefficient is low. However, is this sample the only sample that contains that proteins at all, they are more likely to be in the same complex.
Note: A low sample number increases the chance that two indirectly interacting proteins are in just one (the same) sample. This leads to a reduced sensitivity. The Jaccard coefficient is less meaningful here and highly influenced by the sample selection.

Version of the "CC" function that sets S itself (CC_auto)

Input: *NNN-matrix* where each row i stands for the near neighbor network of protein i

1 Run CC with **S** = 0.01 on *NNN* and store the result in "*CC_current*"
2 Count the number of proteins that are in at least one core complex
3 Search the highest possible value for **S** in a range between 0.01 and 1 where running CC with **S** on this *NNN* leads to the same number of counted proteins as in step 1

Output: *CC*: M x M matrix for all proteins vs. each other. $CC_{i,j}$ is 1 if protein j is in the core complex of protein i and 0 otherwise.

Function for joining core complexes (joinCC)

External parameters:

P: Core complex jaccard coefficient threshold, default is 0.5

Input: *CC* (output from CC)

Note: One row x in *CC* represents the core complex that belongs to the protein x.
1 Create an upper triangle matrix "*toJoin*" which rows and columns represents all core complexes vs. each other
2 For each combination of two row indicies *i,j* from *CC*:
 2.1 Let *I* be a vector with the column indicies of *CC* that contain a 1 in the row *i*, J the same for the row *j*
 2.2 IF the ratio between the number of indicies that are in both vectors ***I*,*J*** and the total number of indicies in the shorter vector is at least **P** THEN set $toJoin_{i,j}$ to 1
3 See *toJoin* as a adjacency matrix of a *graph**
3 Create a list of vectors "*joinedCCs*"
4 For each connected component** in *graph*:
 4.1 Select all core complexes (rows from *CC*) from the current connected component
 4.2 Select all column indicies where *CC* is 1 in at least one of the selected rows and append this vector of column indicies to joinedCCs

Output: *joinedCCs*: A list of clusters, where each cluster stands for a joined core complex and contains the set of proteins that build this complex.

* Let each core complex be a vertex in a graph and let $toJoin_{x,y}$ contain a 1 when an edge exists between vertex x and vertex y. *toJoin* is called the adjacency matrix of this graph.

** A connected component is a maximum set of vertices where each vertex can be reached directly or indirectly from each other vertex in the set.

Function that combines the three steps (4N)

External parameters:

- **U:** co-occurrence threshold (automatically selected or external)
- **C**: Cosine-distance threshold (external)
- **S**: NNN reciprocity threshold for building core complexes (automatic or external)
- **P**: Core complex Jaccard coefficient threshold, default is 0.5

Input: *IP-matrix*

1 run **NNN** on IPmatrix with automatic or given **U** and given **C, P**
2 IF **S** is given THEN run **CC** on the result of **NNN** ELSE run **CC_auto** on the result of **NNN**
3 run **joinCC** with given or default **P** on the result of **CC**

Output: *joinedCCs*: A list of clusters, where each cluster stands for a joined core complex and contains the set of proteins from this cluster

Recursive part of the HC4N method (HC4N_recursive)

External parameters:

- **P**: Core complex Jaccard coefficient threshold, default is 0.5

Input: *IP-matrix* and *the joinedCCs* from a run of **4N**

1 Create a empty list "*clusterTree"*
2 For each cluster *y* in *joinedCCs*:
  2.1 Create a matrix *IP-subset* that contains all rows of the proteins from *y* and all columns where any of the proteins has a non zero value
  2.2 Run **4N** with given **P** on *IP-subset* to create a *joinedCCs-subset*
  2.3 IF *joinedCCs_subset* contains only one cluster THEN
    2.3.1 add a leaf node to the *clusterTree* that contains this cluster
  2.4 ELSE
    2.4.1 RUN **HC4N_recursive** with *IP-subset* and *joinedCC-subset* as input and store the result in "*HC-subset*"
    2.4.2 add an inner node to *clusterTree* that contains *HC-subset* and the cluster *y*
3 return *clusterTree to the precious instance*

Hierarchical clustering using 4N (HC4N)

External parameters:

- **P**: Core complex Jaccard coefficient threshold, default is 0.5
- **C**: Cosine-distance threshold, default is 40
- **U**: Co-occurrence threshold for 4N (automatically selected or external)
- **S**: NNN reciprocity threshold for building core complexes (automatic or external)

Input: *IP-matrix*

1 Run **4N** with the given parameters to create the first level
2 Run **HC4N_recursive** with the IP-matrix and the result of **4N** as input, and with the given parameter **P**
3 Return clusterTree

Output: A tree structure where every node is either a leaf that contains a cluster or an inner node that contains a cluster and has attached children nodes.

Function for joining clusters (joinClusters)

External parameters:

- **P**: Jaccard coefficient threshold, default is 0.5

Input: a set of clusters as result from **HC4N**

1 Collect all clusters from the input
2 Create clusters in the same way as **joinCC** , but with the real Jaccard coefficient as similarity measure instead of "amount of proteins in the smaller cluster which occurs in the larger cluster"

Output: *joinedClusters*: A list of clusters, where each cluster contains a set of proteins
