# Supplementary material for: Towards a Hierarchical Strategy to Explore Multi-Scale IP/MS Data for Protein Complexes
Source: PLoS One. 2015 Oct 8;10(10):e0139704. doi: 10.1371/journal.pone.0139704 (PMC4598013; doi:10.1371/journal.pone.0139704)
Supplement: S2 Text — Usage and parameter selection strategy for HC4N. (PDF) [file pone.0139704.s004.pdf]

## General HC4N strategy

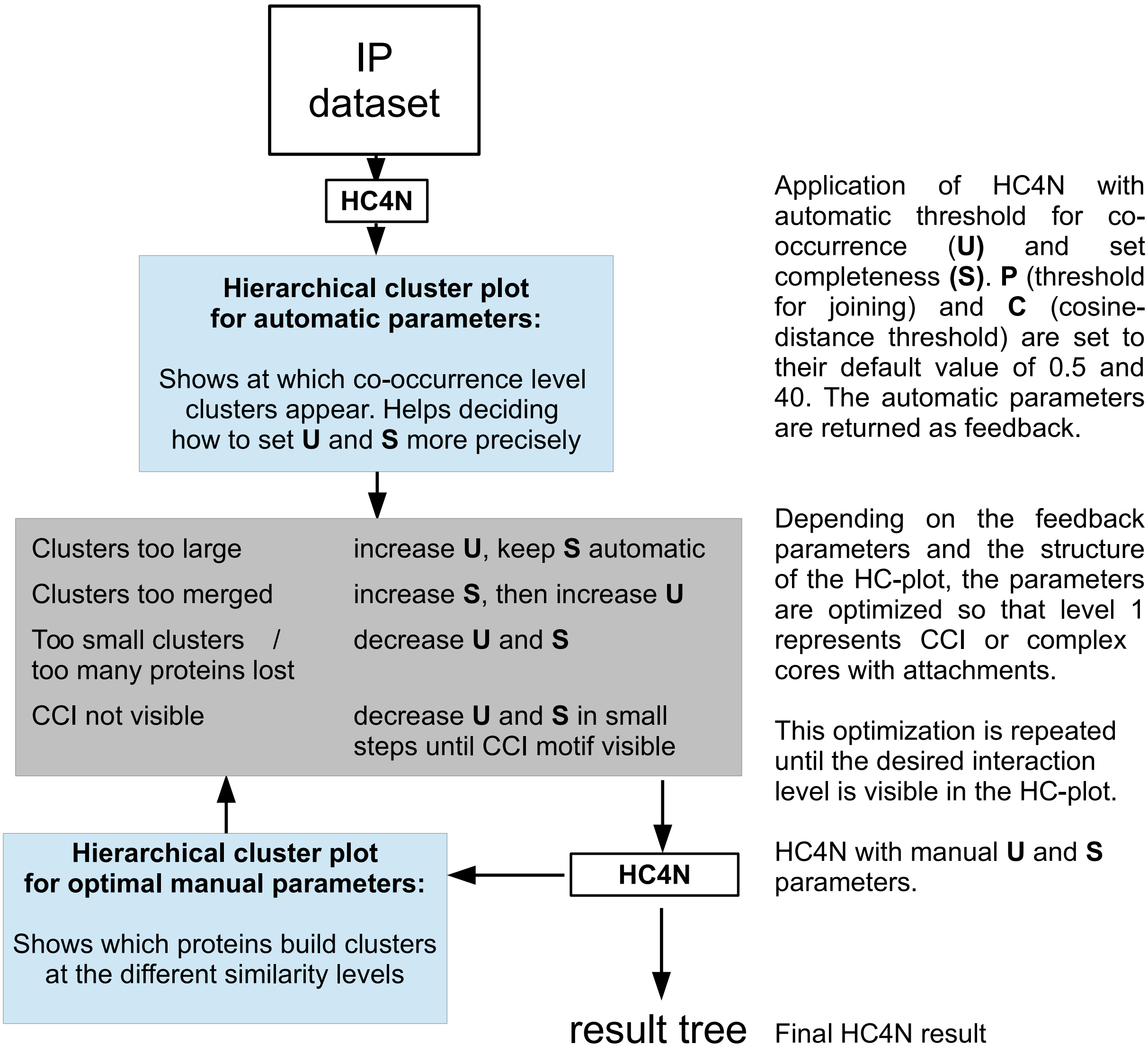


Figure 1: HC4N is applied with automatic parameters for co-occurrence and set completeness. The hierarchical cluster plot is then used as feedback to optimize the parameters for a specific dataset and for a desired interaction level. The parameters are set in a way that HC4N level 1 represents the highest interaction level, for instance, the level of complex–complex interactions or the level of complex cores.
