# Supplementary material for: Towards a Hierarchical Strategy to Explore Multi-Scale IP/MS Data for Protein Complexes
Source: PLoS One. 2015 Oct 8;10(10):e0139704. doi: 10.1371/journal.pone.0139704 (PMC4598013; doi:10.1371/journal.pone.0139704)
Supplement: S3 Text — HC4N analyses on the large–scale datasets. (PDF) [file pone.0139704.s005.pdf]

HC4N analysis on Krogan2004

HC4N is first applied with automatic settings for minimum co-occurrence (U) and setwise completeness (S) and default value (0.5) for P. HC4N sets U to 0.125 and S to 0.64. In the HC-plot, a large cluster covers more than one third of the image. We can conclude from that, that the co-occurrence parameter for the first level is set too low, because it is implausible that all the proteins interact. The HC-plot shows in addition, that the dataset splits into smaller clusters at a U around 0.35.

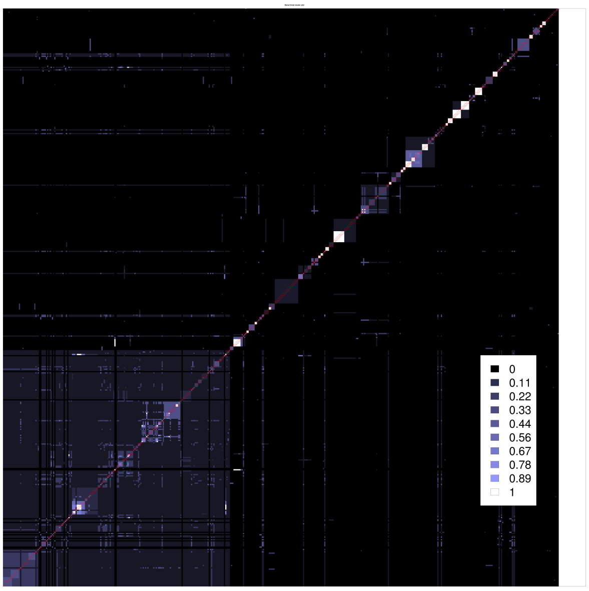

Figure 1: Hierarchical cluster plot for the application of HC4N with automatic/default parameters. Automatic U is 0.125, automatic S 0.64.

HC4N is applied with a U of 0.35 and automatic S. The HC-plot now shows a large amount of separate clusters and also larger clusters with a complex inner structure. This is a sign that the new parameter setup is suitable to better find the different types of complexes in the data.

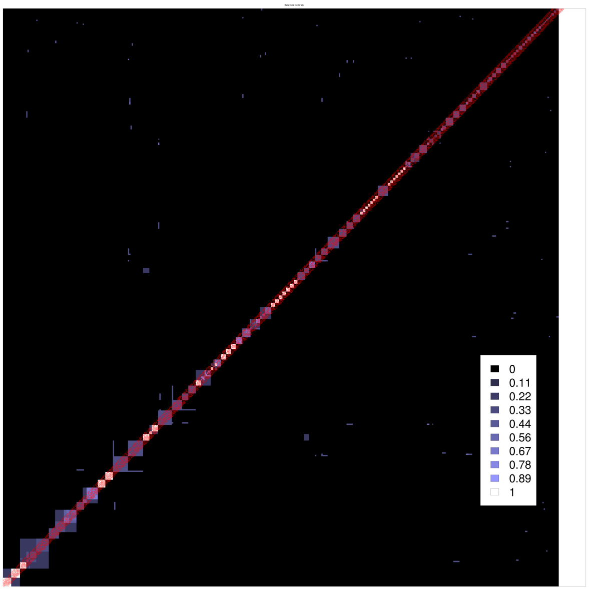

Figure 2: HC-plot for the application of HC4N with the manual U of 0.35 and automatic S.

HC-plots for Krogan2004 with added noise

To examine the reaction of HC4N to noise, random false positive values were added to the dataset. The original dataset contains 1.5% purifications. The number was increased by setting random positions in the IP/MS matrix to 1. HC4N in its automatic mode is applied to the datasets. On data with added noise, the software creates more and smaller clusters with lower co-occurrence. Previously present clusters with high co-occurrence are not visible anymore.

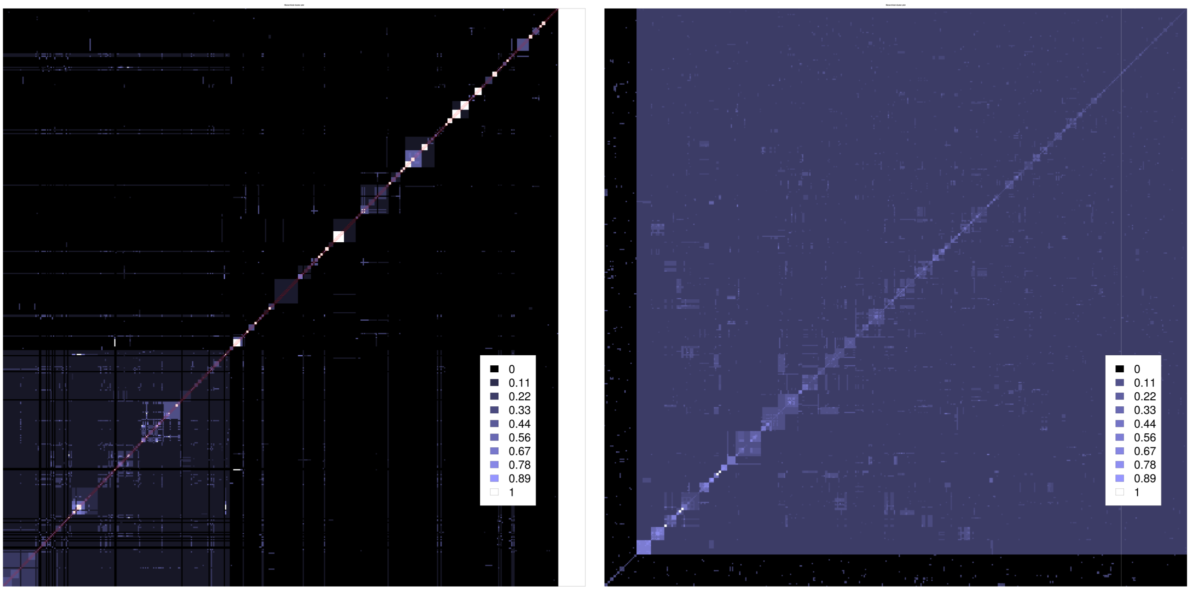

Figure 3: Left: HC-plot from the automatic run on the original Krogan2004 dataset. 388 clusters are found. Right: HC-plot for 1.5% noise added. Approx. 2000 clusters are found.

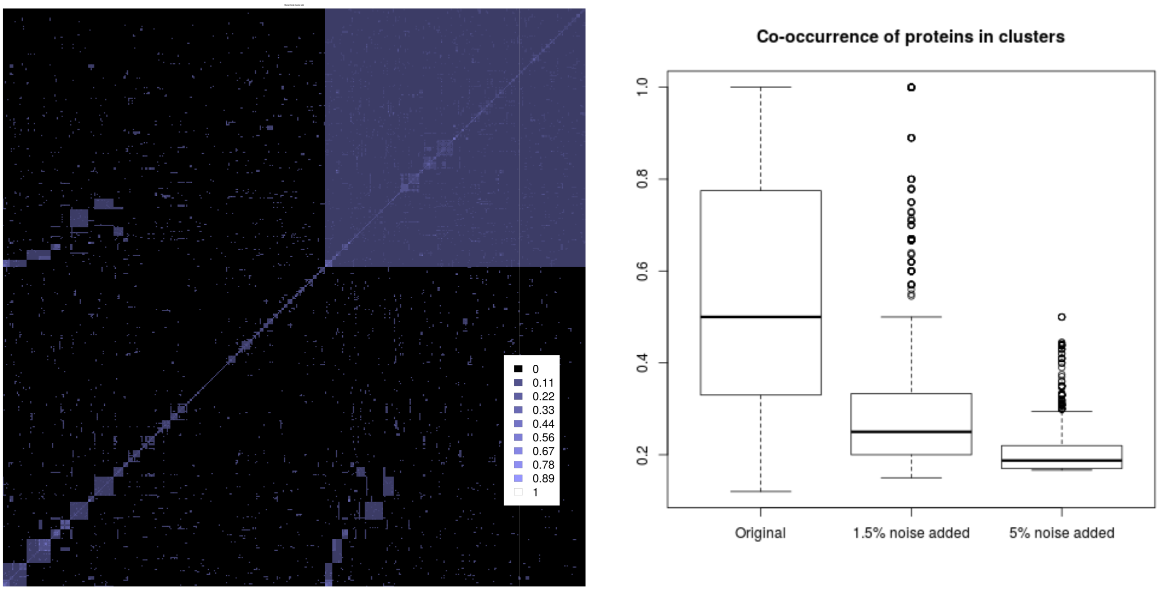

Figure 4: Left: HC-plot for 5% noise added. Approx. 2500 clusters are found. Right: The co-occurrence of the clusters in data with added noise is lower than in the clusters of the original dataset.

HC4N analysis on Gavin2006

HC4N is first applied with automatic settings for U and S and default value (0.5) for P. The HC-plot (only a subset is shown here as the whole plot is hard to interpret) shows one large cluster which falls into smaller clusters at a U of 0.25.

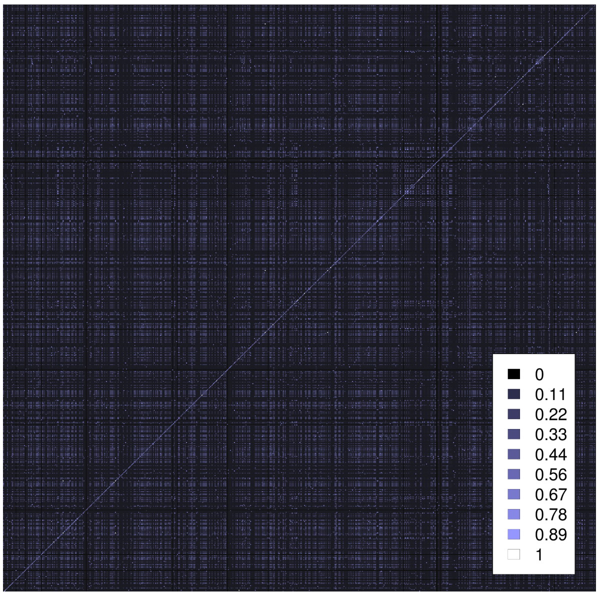

Figure 5: HC-plot (cutout) for the application of HC4N on the results of a HC4N with automatic/default parameters.

HC4N is applied with the U of 0.25 and creates ca. 800 clusters. The new HC-plot (of which only a subset is shown here) shows that HC4N captured clusters here at different levels of co-occurrence.

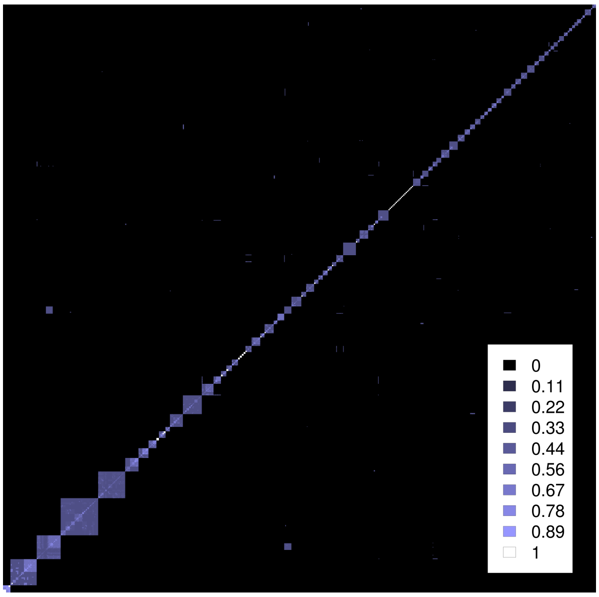

Figure 6: HC-plot (cutout) for the application of HC4N on the results of a 4N with manual parameters.

HC4N analysis on Krogan2006 and Malovannaya

Note: The HC-plots for those analyses are very large and not part of this SM.

Krogan2006

Low average co-occurrence of proteins in this large dataset lead to very low U and S in the automatic 4N. The large clusters start to separate at a U of 0.1. HC4N finds 22000 clusters with co-occurrence values between 0.1 and 1. Many clusters in the result have a very low value and to reduce their number, only the approx. 15400 clusters with a co-occurrence > 0.2 are considered. They were joined with P of 0.7 to eliminate duplicates. Validating of the remaining 6300 with cyc2008 leads to a accuracy of 0.6.

Malovannaya

HC4N with automatic settings is applied to the "Malovannaya" dataset. The automatic U is below a very low 0.05, because some proteins hardly co-occur at all. HC4N is set stricter ($U = 0.2$, $S = 0.25$) and finds 7554 clusters. The clusters are joined with a P of 0.7 to eliminate duplicates. This leads to ca. 5000 clusters which were compared to the reference. The accuracy lies at 0.88, the separation is 0.2.
