# Supplementary material for: Towards a Hierarchical Strategy to Explore Multi-Scale IP/MS Data for Protein Complexes
Source: PLoS One. 2015 Oct 8;10(10):e0139704. doi: 10.1371/journal.pone.0139704 (PMC4598013; doi:10.1371/journal.pone.0139704)
Supplement: S4 Text — Analysis of Gavin2006-SOI and Malovannaya-SOI with Biclust, apComplex, HICLAS. (PDF) [file pone.0139704.s006.pdf]

Analysis of Gavin2006-SOI with Biclust, apComplex, HICLAS

Biclust finds 25 complexes of sizes between 2 and 11 proteins in the dataset. Every cluster contains proteins from only one of the POL complexes, but none of the clusters represents any of the complexes completely. The result is difficult to analyze as the clusters overlap highly and do not contain information why certain protein sets were assigned to clusters. A Figure in the section shows the result of Biclust as overlay on the result network from HC4N to illustrate the situation.
HICLAS is applied for different complex numbers and the residuals are examined. The residuals decline with higher cluster number and an optimal number of clusters is hard to find. We reduced the weight of the negative discrepancies by the factor 5. The new residuals plot shows low residuals at 6 clusters. The results with this cluster number distinguishes between all three POL complexes. The residuals plots and the cluster result can be found in section and .
apComplex finds more than 80 clusters in Gavin2006-SOI. Each of them represents parts of the POL complexes which leads to a very low separation. When the clusters are joined with a threshold of 0.5, 6 complexes remain that largely represent the three POL complexes. Four proteins are misclassified by the algorithm.
In conclusion, the results from HICLAS and apComplex represents the three complexes better than the result from Biclust. However, selecting the right amount of clusters is crucial for HICLAS and the result on only three clusters does not separate the complexes. Biclust suffers from too many and too small clusters, which may be caused by noise in the data.

Biclust result of the Gavin2006-SOI

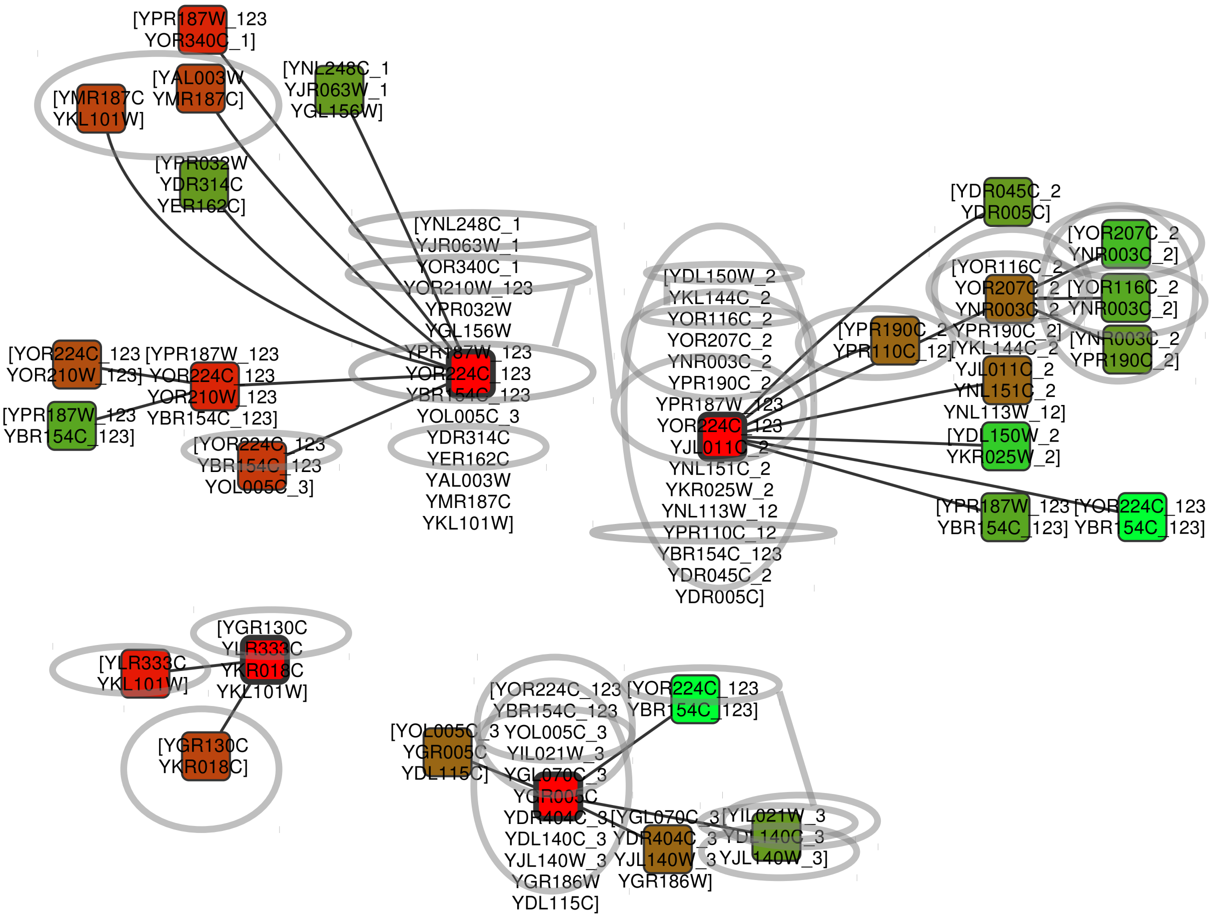

The result from HC4N is overlaid with the result from BICLUST. Each ellipse is one of the clusters from BICLUST. Connected ellipses symbolize one cluster. It is clear that BICLUST creates too much too small cluster, that represent only parts of the complexes.

HICLAS result of the Gavin2006-SOI

Residual plots for the HICLAS analysis:

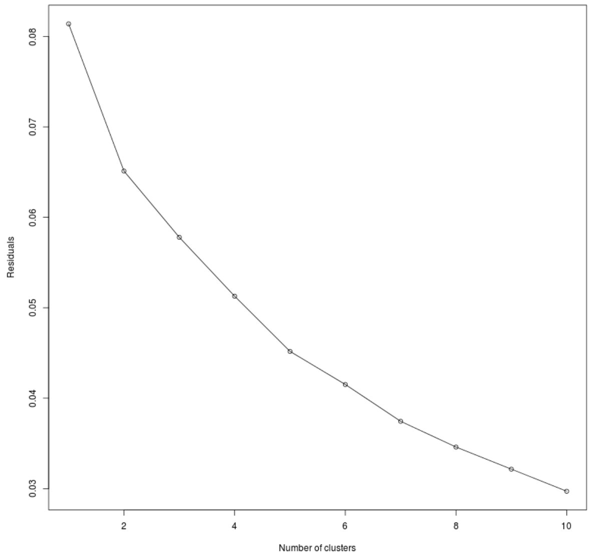

Figure 1: HICLAS was applied with $1 - 10$ clusters. The plot shows the residuals for a HICLAS where positive and negative discrepancies are weighted equally. It does not show a clear decrease anywhere that would make the cluster number selection possible.

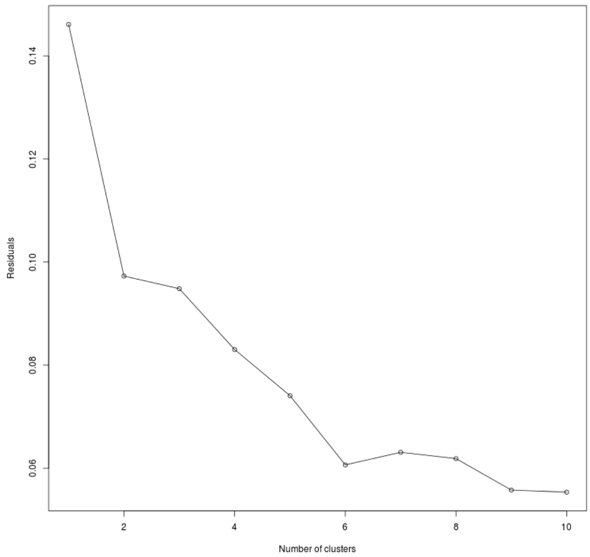

Figure 2: HICLAS was applied again with a decreased weighting for negative discrepancies. The plot shows a clear decrease at 6 clusters. Note that the absolute residuals are lower for the first plot, but in this example, the cluster result is better for the second setting.

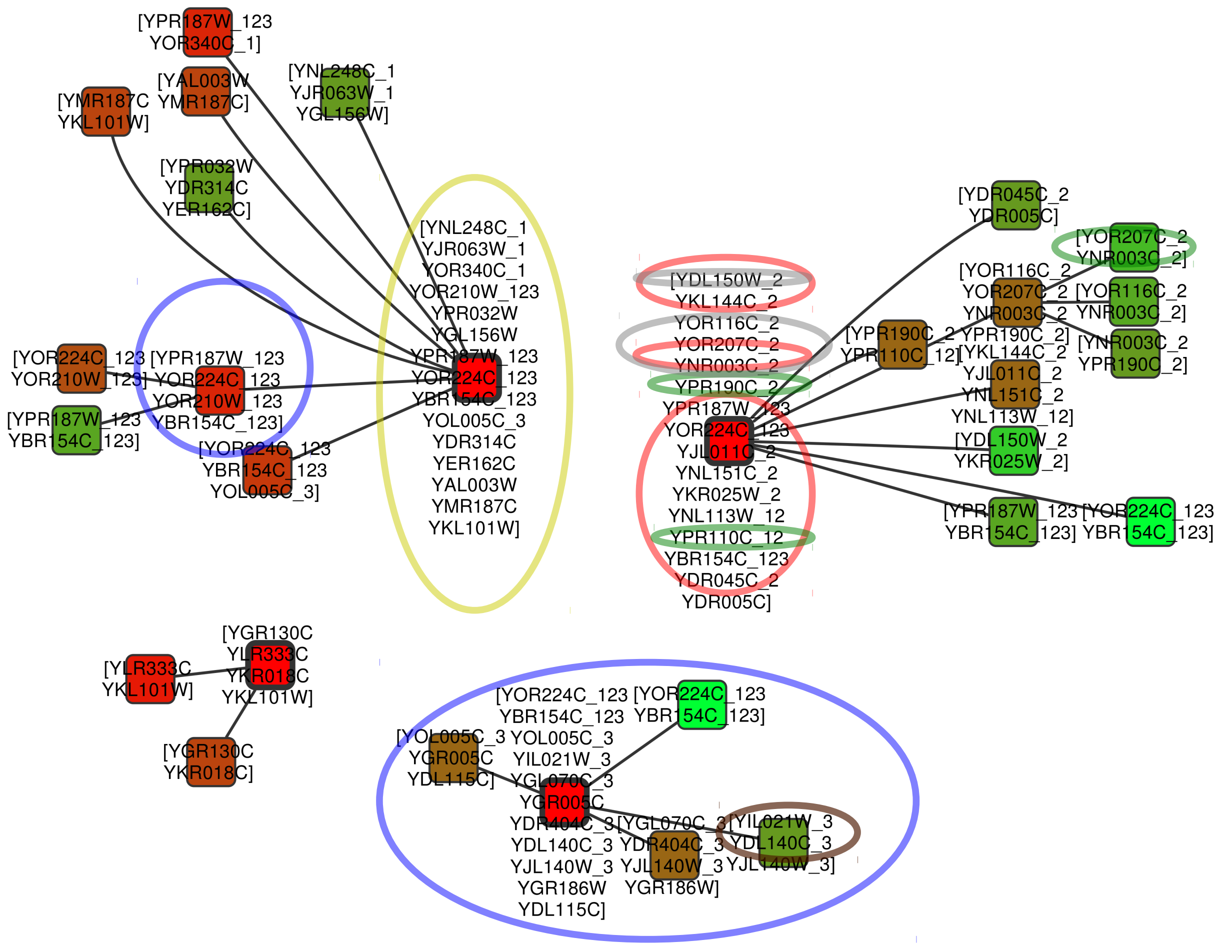

The result from HICLAS overlays the result from HC4N.

Each of the 6 colors ( ) is a cluster. The HICLAS result with 6 complexes represents the three POL complexes largely.

Analysis with HICLAS and apComplex on Malovannaya SOI

Residuals for the HICLAS analysis:

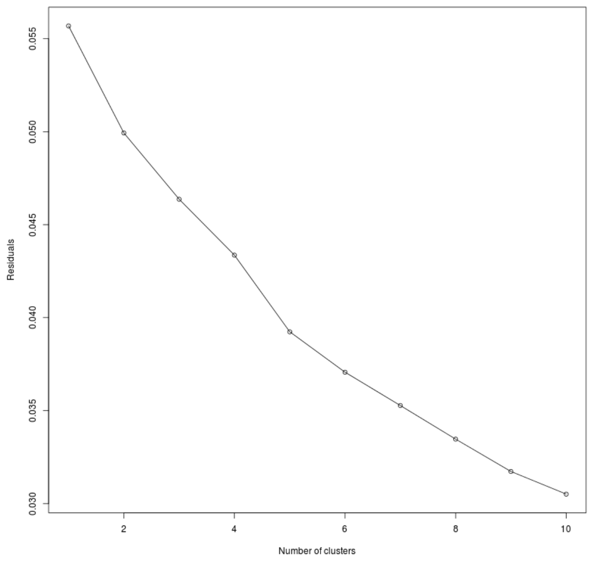

Figure 3: HICLAS applied with $1-10$ clusters and equally weighted discrepancies. The optimal number of clusters is not clear.

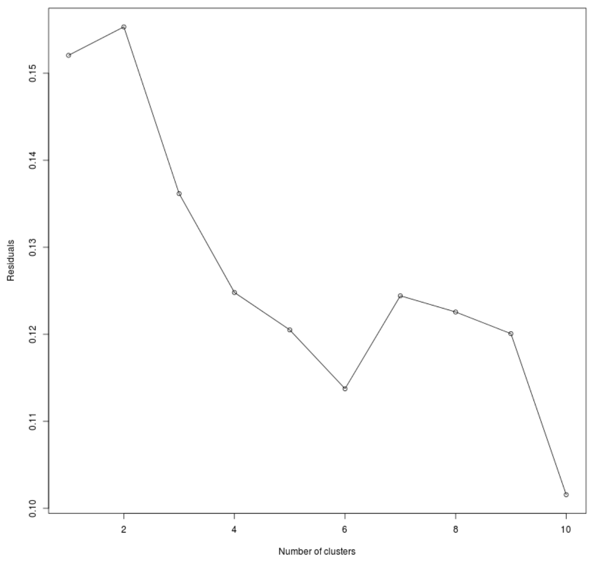

Figure 4: Residuals for HICLAS with lower weighted negative discrepancies. The residuals show that 6 clusters lead to relatively low residuals.

HICLAS is applied with 1-10 complexes. The residuals plot shows linearly decreasing residuals when discrepancies are equally weighted. The negative discrepancy weight is reduced by the factor 10 and we obtained a residuals plot that shows a clear decrease at 6 clusters. In this result, HICLAS finds INT together with the connection protein POLR2A and the PPPase proteins. The complex around CDK9/MLLT is found together with INT and POL proteins, which is a false classification as the complex is close to the MED complex. The MED complex itself is predicted completely. The POL complex is found correctly, together with the proteins around GPN1. The proteins around ZBF592 are found completely, together with one of the INT proteins. Concluding, HICLAS can largely infer the complexes, but it fails to separate POL and INT and some proteins are misclassified.
apComplex finds over 7000 complexes in ”Malovannaya-SOI”. After joining the complexes with a threshold of 0.5, only 50 complexes remained. A accuracy of 0.69 is reached, at a high sensitivity of 1 but a low PPV and low separation.
