# Supplementary material for: Towards a Hierarchical Strategy to Explore Multi-Scale IP/MS Data for Protein Complexes
Source: PLoS One. 2015 Oct 8;10(10):e0139704. doi: 10.1371/journal.pone.0139704 (PMC4598013; doi:10.1371/journal.pone.0139704)
Supplement: S5 Text — HC4N result graph for the Malovannaya-SOI analysis. (PDF) [file pone.0139704.s007.pdf]

HC4N result graph of the Malovannaya SOI

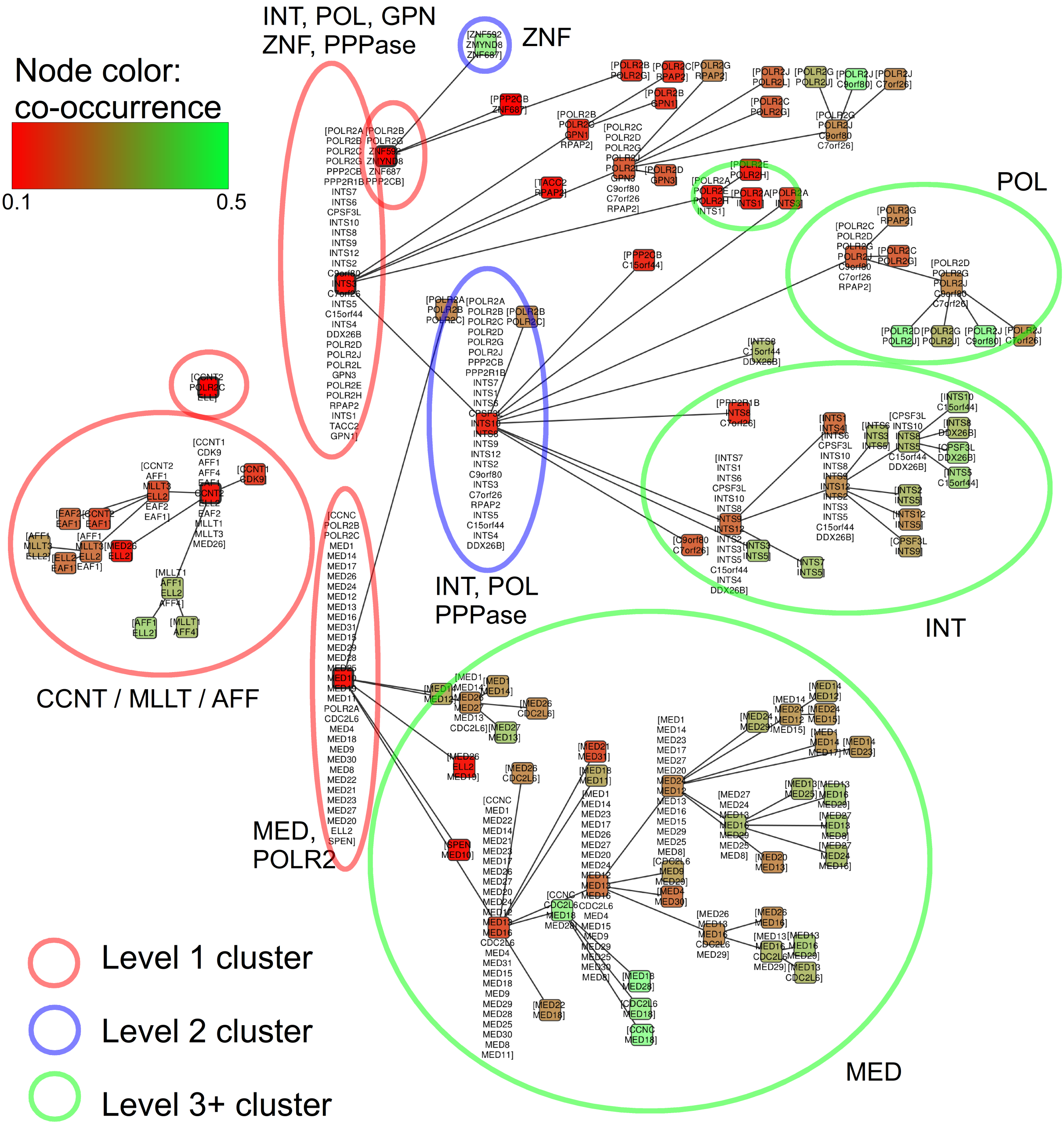

Each node is a cluster found by HC4N. The circles show which complexes from the reference were covered by which nodes. Complexes can share proteins and children complexes can appear multiple times. The POL-proteins at the top of the Figure are not circled for this reason. The graph shows that in higher interaction levels, MED/POL build a complex and as well MED/INT. The shared proteins are the POLR proteins. On a lower interaction level, INT, MED and POL are separated correctly and it is visible from the similarity levels, that INT and POL are interacting more closely than MED and POL. The CCNT/MLLT/AFF contains a MED protein as indicator for their possible interaction with MED. GPN is shown to be close to POL, as well as ZNF.
