# Supplementary figures and images for: Towards a Hierarchical Strategy to Explore Multi-Scale IP/MS Data for Protein Complexes
Source: PLoS One. 2015 Oct 8;10(10):e0139704. doi: 10.1371/journal.pone.0139704 (PMC4598013; doi:10.1371/journal.pone.0139704)

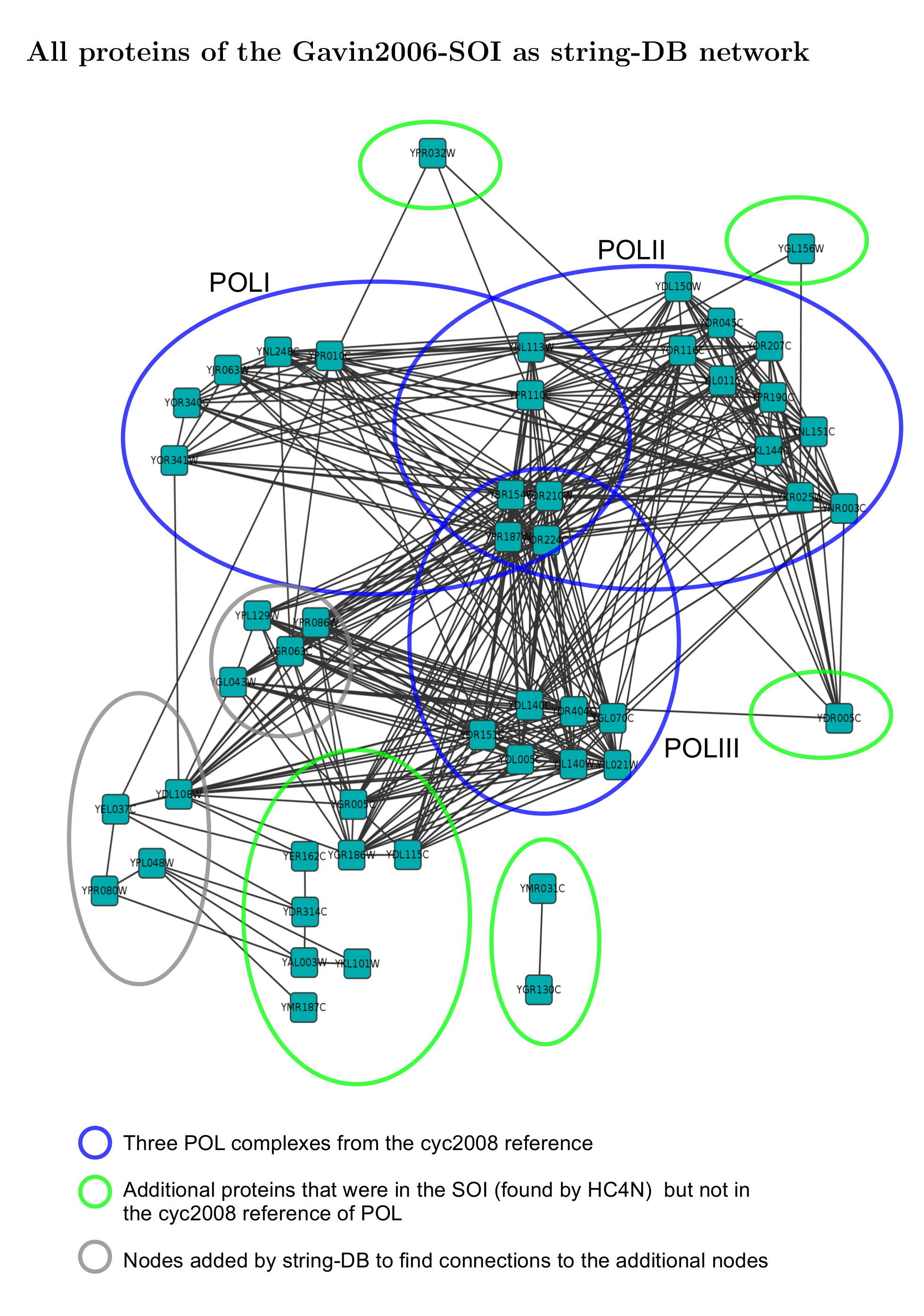

Supplement: S1 Fig — All proteins of the Gavin2006-SOI dataset as string-DB network. (TIFF) [file pone.0139704.s008.tiff]
